# Investigating unexplained genetic variation and its expression in the arbuscular mycorrhizal fungus *Rhizophagus irregularis*: A comparison of whole genome and RAD sequencing data

**DOI:** 10.1371/journal.pone.0226497

**Published:** 2019-12-27

**Authors:** Frédéric G. Masclaux, Tania Wyss, Marco Pagni, Pawel Rosikiewicz, Ian R. Sanders

**Affiliations:** 1 Department of Ecology and Evolution, University of Lausanne, Switzerland; 2 Vital-IT Group, Swiss Institute of Bioinformatics, Switzerland; Institute for Sustainable Plant Protection, C.N.R., ITALY

## Abstract

Arbuscular mycorrhizal fungi (AMF) are important symbionts of plants. Recently, studies of the AMF *Rhizophagus irregularis* recorded within-isolate genetic variation that does not completely match the proposed homokaryon or heterokaryon state (where heterokaryons comprise a population of two distinct nucleus genotypes). We re-analysed published data showing that bi-allelic sites (and their frequencies), detected in proposed homo- and heterokaryote *R*. *irregularis* isolates, were similar across independent studies using different techniques. This indicated that observed within-fungus genetic variation was not an artefact of sequencing and that such within- fungus genetic variation possibly exists. We then looked to see if bi-allelic transcripts from three *R*. *irregularis* isolates matched those observed in the genome as this would give a strong indication of whether bi-allelic sites recorded in the genome were reliable variants. In putative homokaryon isolates, very few bi-allelic transcripts matched those in the genome. In a putative heterokaryon, a large number of bi-allelic transcripts matched those in the genome. Bi-allelic transcripts also occurred in the same frequency in the putative heterokaryon as predicted from allele frequency in the genome. Our results indicate that while within-fungus genome variation in putative homokaryon and heterokaryon AMF was highly similar in 2 independent studies, there was little support that this variation is transcribed in homokaryons. In contrast, within-fungus variation thought to be segregated among two nucleus genotypes in a heterokaryon isolate was indeed transcribed in a way that is proportional to that seen in the genome.

## Introduction

Arbuscular mycorrhizal fungi (AMF) colonize the roots of the majority of land plants, improving plant nutrient and water uptake in exchange for plant-assimilated carbohydrates and lipids [1, 2]. Arbuscular mycorrhizal fungi form hyphae and spores that contain a continuous cytoplasm with many co-existing nuclei. Unlike other fungi, there is no known stage during the AMF life cycle in which only one nucleus initiates a new generation. This suggests that the co-existence multiple nuclei in the AM fungal cytoplasm may be important for fungal growth and cellular functions [3].

Over the last two decades there has been some debate about whether AMF are homokaryons (possessing genetically identical nuclei) or heterokaryons (possessing two or more genetically different nuclei). The ensuing, and lengthy, debate about whether or not AMF are heterokaryons has largely confounded two separate questions. 1. Are AMF heterokaryons or homokaryons? The definition of a heterokaryon is the co-existence of 2 or more genetically different nuclei which is purely qualitative and not dependent on whether the nuclei display a large number of differences or a small number. 2. If AMF are heterokaryons, how much genetic variation within an AMF isolate is distributed among nuclei? This second question is quantitative but only concerns the heterokaryotic state. Resolving this issue of genetic variation in AMF is biologically highly relevant. For example, clonally produced single spore offspring of the AMF species *R*. *irregularis* show high variation in their phenotype [4] and extremely high variation in their effects on plant growth [5]. Such variation in clonally produced *R*. *irregularis* could potentially be due to heterokaryosis and the differential inheritance of different nucleus genotypes among offspring.

**Fig 1:**
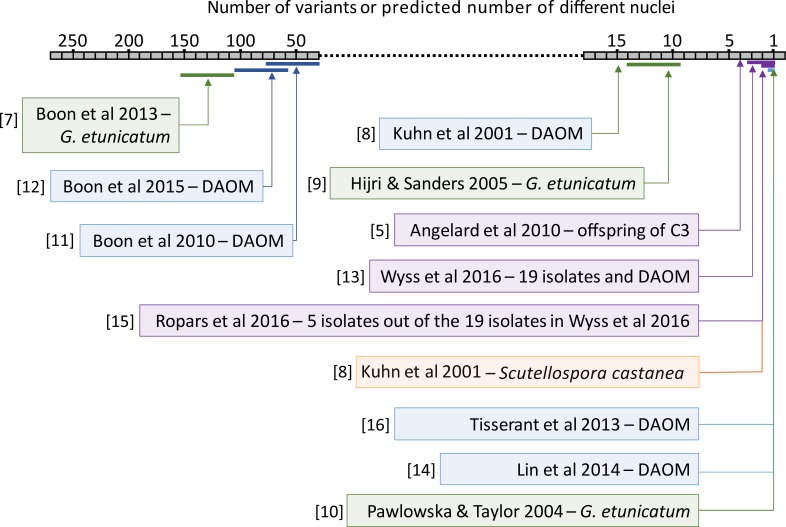
Range of reported levels of intra-isolate genetic variation in different AMF species and different studies. The level of intra-isolate genetic variation is expressed as the number of alleles observed per position in the genome or the predicted number of genetically different nuclei per AMF isolate on the grey horizontal bar. Levels of intra-isolate genetic variation were determined by sequencing methods. Green represents *Glomus etunicatum* although the studies did not use the same isolates. Blue represents *Rhizophagus irregularis* isolate DAOM 197198. Purple represents studies including isolates of *R*. *irregularis* other than DAOM 197198. It is important to note that within some studies a greater number of alleles was observed compared to the number of different nuclei predicted, owing to assumptions about ploidy or gene copy number. References cited in the figure that are not cited in the manuscript text: [7–10]. Methods used in referenced studies: [9–11] sanger sequencing; [7] amplicon pyrosequencing; [12] whole genome and amplicon pyrosequencing; [5] amplicon fluorescence and amplicon pyrosequencing; [13] ddRAD-seq and Sanger sequencing; [8] Sanger sequencing and fluorescent *in situ* hybridization; [14] WG amplification and Illumina® WG sequencing; [15] Illumina® WG sequencing; [16] Sanger, Illumina®, pyro- and Pacific Biosciences sequencing.

Studies over the last two decades that have tried to address the two questions outlined above are highly inconsistent in their conclusions regarding the amount of genetic variation existing within an AMF isolate and the prediction of the number of genetically different nuclei (summarized in Fig 1). However, there has been a lack of studies focused on the same species or isolate where sequence data can be directly compared. Some species studied probably diverged from each other hundreds of millions of years ago, making comparisons between some of these studies as largely meaningless [6]. Furthermore, different approaches have been used as technologies have developed over the years.

More recently, studies using different techniques and sequencing platforms have concentrated on describing the genome of one haploid species of AMF, *R*. *irregularis*, and its within and among isolate variation [13, 15–18]. Thus, there are now directly comparable data originating from independent studies. The whole genome of the *R*. *irregularis* isolate, DAOM197198, was sequenced showing that within fungus polymorphism existed but was low. The authors concluded that this isolate was a homokaryon [14, 16] and did not try and explain the variation. Double-digest restriction site-associated sequencing (ddRAD-seq) was performed on DAOM197198 and 19 other *R*. *irregularis* isolates [13], showing different levels of within-fungus polymorphism among the isolates (ranging from a SNP density in single-copy coding regions of around 1 SNP Kb^-1^, which was similar to DAOM 197198, to approximately 2.8 SNPs Kb^-1^. This suggested the occurrence of isolates with a predominantly homokaryon or heterokaryon state; where heterokaryons comprised predominantly only two different genotypes of nuclei, but with the possibility of additional genetically different nuclei at low frequency. Ropars *et al*. (2016) sequenced the whole genome of five of the 19 *R*. *irregularis* isolates and concluded that three of these isolates; A1, B3 and C2 were homokaryons and that two isolates; A4 and A5 were heterokaryons with two genetically different nucleus genotypes [15]. Most notably in that study, the authors defined homokaryon isolates and dikaryon isolates based on the presence of either one or two alleles at a putative mating type locus and not solely the basis of allele frequencies at bi-allelic sites. Notably, none of these studies have recorded as high levels of polymorphism in *R*. *irregularis* as those observed by Boon et al. [11, 12] (Fig 1).

All studies revealed a level of polymorphism unexplainable by a strict homokaryon state [13, 15, 16] [14]. In each case, bi-allelic sites existed in isolates that had been predicted to be homokaryons. However, this variation was largely discounted as being due to sequencing errors. Plots of allele frequency at bi-allelic sites in proposed homokaryons and heterokaryon isolates revealed a peak at 0.5 in several isolates but with additional variation at other frequencies[13]. A clear 0.5 peak of frequency at bi-allelic sites is expected for a heterokaryon with equal proportions of two nucleus genotypes or a diploid. Ropars et al. (2016) found a peak at 0.5 frequency in A4 and A5, but a U-shaped distribution in the other isolates, the interpretation of which is difficult to explain [13, 15].

Wyss et al. (2016) observed within-fungus genetic variation among biological replicates of the same isolates, thereby, reducing the probability that the variation was the result of sequencing error [13]. Ropars et al. (2016) also constructed replicate libraries for whole genome sequencing, although they pooled them for further analysis, and did not use them as replicates[15]. We hypothesized that bi-allelic sites observed in the genome of a given isolate in a replicated study, and between the two independent studies, will be consistent if they did not arise from sequencing error. Thus, observed variation represents true variation and is not due to sequencing error. Using the replicates of Wyss et al. and the data from Ropars et al., treated as replicates, allows to test whether the unexplained variation in these studies is indeed real biological variation or an artefact of sequencing. This was the first goal of this study.

So far there has been almost no attention paid to the functional significance of any within-fungus genome variation observed in AMF. However, single spore offspring of clonally produced *R*. *irregularis* shows a high degree of variation in their phenotype and their effects on plant growth [4, 5]. Transcriptome profiling of *R*. *irregularis* would reveal whether the genome variation observed at bi-allelic sites is transcribed or whether unequal or single-allele expression occurs. However, as yet, no studies exist in AMF relating variation at the transcriptome level back to variation observed at the genome level. The detection of bi-allelic transcripts that match observed variation in the genome would confirm the existence of bi-alleles in the genome and would indicate that the variation could potentially have consequences on the phenotype. Expression of two alleles of a given gene, unequal expression of the two alleles, or expression of only one of them, could all have consequences on the phenotype of the fungus or its effect on plant growth. If alleles were truly located on two genetically different nuclei, it would mean that it would also represent a measure of the contribution by the two different AMF nucleus genotypes to overall gene expression in the fungus. In plants, the contribution of two different genomes (for example in hybrids or polyploids) is often highly unequal and the contribution from each genome can be tissue specific [19]. Thus, the existence of two different genotypes of nuclei does not necessarily mean that gene expression will be in equal proportions from the two alleles at a given locus. Thus, the second goal of our study was, therefore, to investigate whether variation observed in the *R*. *irregularis* transcriptome matched variation observed in the genome as an additional test of whether the variation really exists. Additionally, we wanted to know the contribution of each nucleus genotype to overall gene expression in this fungus.

We, therefore, used the available genome data on *R*. *irregularis* to compare within-isolate genome polymorphism in *R*. *irregularis* isolates A1, A4, A5, B3 and C2 as recorded by whole genome sequencing and dd-RAD-seq and test whether the unexplained variation in these studies is indeed real biological variation or an artefact of sequencing. We also conducted transcriptome profiling on the isolate C3 and C2 to see if variation found at the genome level is mirrored at the transcriptome level, i.e. whether this variation is co-expressed, and in what proportion the alleles are expressed. Isolate C3 was shown by Wyss et al. 2016 to be a clone of A4 that was predicted by Ropars et al. 2016 to be a heterokaryon with two nucleus genotypes [15] [13]. The isolate C2 was predicted to be a homokaryon [15]. We conducted *de novo* transcript assembly without using the reference genome assembly so that any observed bi-allelic sites found in both studies were independently discovered and were not biased by the genome assembly. Although heterokaryon can refer to the co-existence of two or more genetically distinct nuclei, here we refer to heterokaryon isolates as those possessing primarily two genetically different nuclei. An alternative term is a dikaryon but we have not used this term in this study because there is a lack of agreement among mycologists regarding whether an AMF species possessing two genetically different nucleus populations fits with the definition of a dikaryon as used for a taxa representing the fungal group referred to as the Dikarya.

## Materials and methods

### Fungal material, RNA extraction and library preparation

Three *R*. *irregularis* isolates (C2, C3 and DAOM197198) were cultured *in vitro* with *Ri* T-DNA transformed carrot roots [20] and a split plate system [21]. Fungal material was extracted from the medium in citrate buffer (450 mL ddH_2_O, 8.5 mL 0.1 N citric acid, 41.5 mL 0.1 N Na citrate) for 20 min and washed with sterile double-deionized water (ddH_2_O). Material from three Petri plates was pooled per sample. There were three samples per isolate. RNA was extracted with the RNeasy Plant Mini kit (Qiagen). Two micrograms of RNA from each sample was used to prepare libraries for RNA sequencing (RNA-seq) using the TruSeq Stranded Total RNA Library Prep Kit (Illumina) with a PCR enrichment step of 15 cycles. The libraries were pooled and sequenced using Illumina HiSeq 2500 (100 bp paired-end reads). Sequences were deposited in the NCBI SRA database (BioProject Accession Number: PRJNA509102).

### All data used in this study

The origin and characteristics of whole genome (WG), ddRAD-seq and RNA-seq data from *R*. *irregularis* and other species that was used in this study is summarized in S1–S3 Tables. The name of the isolate “DAOM197198” is shortened to “DAOM” in some of the figures.

Additionally, in order to find examples to test the RNA-seq KisSplice pipeline (described below), we screened the NCBI sequence archive to select RNA-seq data of species that have homokaryon and heterokaryon states or have homozygote and heterozygote states (S3 Table). The idea was to find species where both states exist like *Arabidopsis* homozygote (*Col*-0 accession) and *Arabidopsis* heterozygote (Hybrid line). Some other fungal species with known homokaryon and heterokaryon states were also included (S3 Table).

### Processing sequence data

Raw reads were processed with the script Tagcleaner.pl to trim Illumina adapters [22]. Reads were processed with PrinSeq-lite.pl version 0.20.4 [23] to remove reads containing ‘Ns’, to trim low quality 3’-ends and retain reads longer than 50 bp. A summary of the read size after filtering is in S4 Table.

### *In silico* prediction of ddRAD-seq fragments

To compare the same sites in ddRAD-seq and WG data, analysis was restricted to ddRAD-seq fragments existing in WG data. I*n silico* digestion with *EcoR*I and *Mse*I was performed on each genome assembly to define predicted ddRAD-seq fragments longer than 49 bp and delimited by a *EcoR*I site and a *Mse*I site. Predicted fragments were aligned to their respective genome with Novoalign v3.02.00 (Novocraft-Technologies, 2014). Fragments that could not be re-aligned were removed. Re-aligned predicted fragments define regions that were considered in subsequent analyses. We refer to ‘predicted ddRAD-seq fragment’ as one of these fragments and to ‘predicted ddRAD-seq region’ to designate the location where the fragment mapped (S1 Fig).

### Identification of interspersed repeats and repeated elements

We use the term ‘repeats’ to include the interspersed repeats (mobile elements and transposon-like regions) and repeated elements (multicopy genes and duplicated regions). The interspersed repeat families were predicted *de novo* with the program RepeatModeler Open-1.0.8 (http://www.repeatmasker.org) and interspersed repeats were annotated with RepeatMasker Open-4.0.6 (http://www.repeatmasker.org).

Every method of repeat detection has its own stringency, which has direct consequences on the number of bi-allelic positions that can be discovered. We wanted to test that identification of poly-allelic sites was not strongly biased by the method of repeat detection. Thus, we used three methods to identify repeated elements to test the influence of underestimating or overestimating the repeated elements on within-isolate polymorphism.

Method M03 was used previously [13]. The *in silico* predicted ddRAD-seq fragments were submitted to pairwise comparisons using ggsearch36; a global pairwise alignment algorithm from the package fasta-36.3.5e [24]. This was done to identify globally similar predicted fragments among the ddRAD-seq fragments. Fragments having a match with another fragment were labelled ‘repeated elements’. The M03 repeats corresponded to interspersed repeats and M03 repeated elements. Method M03 is the default method used in this study (S1 Fig).

Method M07 had a larger spectrum than M03. *In silico* predicted ddRAD-seq fragments were aligned to the whole reference genome using glsearch36 (package fasta-36.3.5e) to identify fragments matching more than one time in the reference genome. Fragments with more than one match were labelled ‘repeated elements’. The M07 repeats corresponded to a combination of interspersed repeats and M07 repeated elements. It is the most stringent of the three methods.

With a third method, M12, *in silico* predicted ddRAD-seq fragments were mapped to the genome assembly with Novoalign V3.02.00 using parameters -r All 100 -R 400 -s0. Fragments with more than one match in the assembly were labelled ‘repeated elements’. The M12 repeats were a combination of interspersed repeats and M12 repeated elements. This method is of medium stringency.

### Gene prediction in genome assemblies

Coding regions were defined with the *ab initio* gene predictor Augustus version 3.1 using the gene model previously defined for the N6 assembly, constructed for the DAOM197198 isolate [13].

### Mapping of WG and ddRAD-seq sequence data on reference genomes

Wyss et al. (2016) mapped reads to the N6 single nucleus DAOM197198 genome assembly published by Lin *et al*. (2014) because other genome assemblies of *R*. *irregularis* isolates were not available at the time [13, 14]. For this study, we used genome assemblies of isolates A1, A4, A5, B3 and C2 for mapping [15]. We aligned reads against the genome assemblies with Novoalign v3.02.12 (Novocraft-Technologies, 2014). The correctly mapped paired-end reads were selected for subsequent analyses using Samtools version 1.3 [25]. Single nucleotide polymorphisms (SNPs), insertions and deletions (indels) and multiple nucleotide polymorphisms (MNPs) were called for each sample using Freebayes version 1.0.2 [26] at positions with a minimum 10× depth of coverage. Only alleles with frequencies greater or equal to 0.1 were recorded. Variant files were filtered to keep the positions with a phred-scaled quality score greater or equal to 30. For every sample, and at each site, we reported whether there was a single allele, two alleles or more than two alleles. A missing value (NA) was assigned if the depth of coverage was below 10×. Because the objective of the study was to study all the within-isolate polymorphism, no filter for the allelic ratio was applied to a position to call it bi-allelic or poly-allelic position.

We compared variants in ddRADseq data [13] and WG data [15]. If the same variants are found among replicates and between two independent studies, then it is highly unlikely that the observed variants are artefacts caused by error generated in the sequencing. Wyss et al. (2016) included 3 replicates per isolate. Ropars et al. (2016) assembled genomes of *R*. *irregularis* that were pooled samples of different libraries that can also be treated as replicates. The numbers of replicates in WG sequencing was between 2 and 3, depending on the isolate.

### Bi-allelic site density and allele frequency

In order to only consider variants in single copy genes, the variant file of each sample was filtered to only retain positions with two alleles in non-repeated and coding regions. The number of bi-allelic positions was divided by the number of positions from predicted ddRAD-seq regions that were sequenced with a coverage greater or equal to 10.

All positions from the variant file were included except those in repeated regions defined by RepeatModeler/RepeatMasker and with less than 10× coverage. The threshold was altered to test the effect of depth of coverage on allele frequency distribution. The list of all allele frequencies was plotted using the density function from the R package ggplot2.

### Depth of coverage and predicted ddRAD-seq regions

Low coverage in samples would limit the ability to reliably detect polymorphisms. Samtools version 1.3 [25] was used to calculate depth of coverage in ddRAD-seq and WG data. Samtools and custom perl script were used to calculate the mean depth of coverage per predicted ddRAD-seq region. A mean coverage greater, or equal to, 10 was necessary to consider a predicted ddRAD-seq region as covered. A similar method was used to calculate the depth of coverage per position required to calculate bi-allelic position density.

### Identification of common bi-allelic positions

The first goal of this study was to compare whether the same bi-allelic sites were found among replicates and studies. The R package UpSetR [27] was applied on lists of bi-allelic positions in non-repeated, coding regions to find common bi-allelic positions in different replicates and generate UpSet plots.

### Discovery of bi-allelic SNPs in RNA-seq data

KisSplice performs a local assembly of RNA-seq reads and detects variants directly from a De Bruijn graph, independently of a reference genome [28]. The methodology to analyse RNA-seq data is summarized in S2 Fig. Because KisSplice reports a very local context around SNPs, we generated reference full-length transcriptome assemblies from RNA-seq data with Trinity [29]. Tritnity v2.3.2 was run with standard parameters (except—SS_lib_type RF). Predicted ORFs were identified in the Trinity assembled transcripts with TransDecoder (v5.0.2). Next, we identified full-length, or nearly full-length, transcripts (https://github.com/trinityrnaseq/trinityrnaseq/wiki/Counting-Full-Length-Trinity-Transcripts, accessed on July 2018). The assembled transcripts were compared to known proteins in Swissprot UniProtKB proteins (www.uniprot.org, accessed on June 2017). The comparison was performed with BLASTX (2.6.0+) with the following parameters: -evalue 1e-20 -max_target_seqs 1 -outfmt 6. The length of the top hit and percentage of the hit length, included in the alignment to the Trinity transcript, were added to the BLASTX result file with the provided script analyze_blastPlus_topHit_coverage.pl. Next, a custom Perl script was applied on the resulting file to keep only the best isoform of each gene and to remove genes with multiple groups (following Trinity’s specifications for ‘group’ and ‘isoform’). KisSplice version 2.4.0 was run on RNA-seq data of each organism with the parameters:—experimental -s 2 -k 41. We focused on the ‘Type0a bubbles’ (KisSplice nomenclature), that are two identical short sequences differing by a single SNP at a given position. Bubble sequences were positioned on the Trinity-reference transcriptome using BLAT with the option -minIdentity = 80. To predict amino acid changes of a SNP, the program KisSplice2RefTranscriptome (K2RT) was run taking as input the predicted ORFs, the Type0a bubbles produced by KisSplice, and the mapping results of BLAT. A custom Perl script removed bubbles that matched two sequences in the reference transcriptome and those with mapping ambiguity. The script also removed bubbles with a read coverage less than six and bubbles with one of the two alleles having less than two observations. Bubbles matching a transcript without a BLASTX hit were removed. These filtering steps ensured that sets of SNPs had the highest possible quality. The KisSplice/K2RT pipeline reports counts of both alleles at bi-allelic positions. Using these values, the distribution of allele frequencies at bi-allelic positions was plotted for each organism.

We tested the pipeline on RNA-seq data generated from different species with known ploidy or heterokaryon/homokaryon status to demonstrate the pipelines efficiency and robustness. The pipeline was applied on *R*. *irregularis* RNA-seq data to evaluate within-isolate polymorphism in RNA transcripts.

### Analysis of bubbles and bi-allelic transcripts

Transcript and bubble sequences were mapped to the reference genome with BLAT using the parameter -minIdentity = 90. The alignments were parsed to quantify the proportion of uniquely mapped sequences. In the case of isolate C3, we used the reference genome of isolate A4, which was previously shown to be a clone of C3.

To investigate whether bi-allelic positions discovered in RNA-seq data matched to bi-allelic sites in the genome, bubble sequences containing 2 alleles were mapped to the reference genome to provide the genomic coordinates of the bi-allelic positions. Next, allelic composition of each bi-allelic position in bubbles was compared to the allele composition of the position in the WG data. Any difference in allele composition was recorded as “inconsistent”. An “inconsistent” position is a bi-allelic position detected in RNA-seq that was not bi-allelic in WG data or that had a different allele composition to the WG data. This could occur because of sequencing error or could be an artefact arising from *de novo* transcript assembly that is independent from a reference genome.

## Results

### Level of within-isolate polymorphism depends on the identity of the reference genome

The identity of the reference genome indeed had a strong impact on the density of poly-allelic positions (S3 Fig), with the lowest density when ddRAD-seq data were mapped onto the respective genome assembly.

### Variants in ddRAD-seq and WG occurred at the same positions

**Fig 2:**
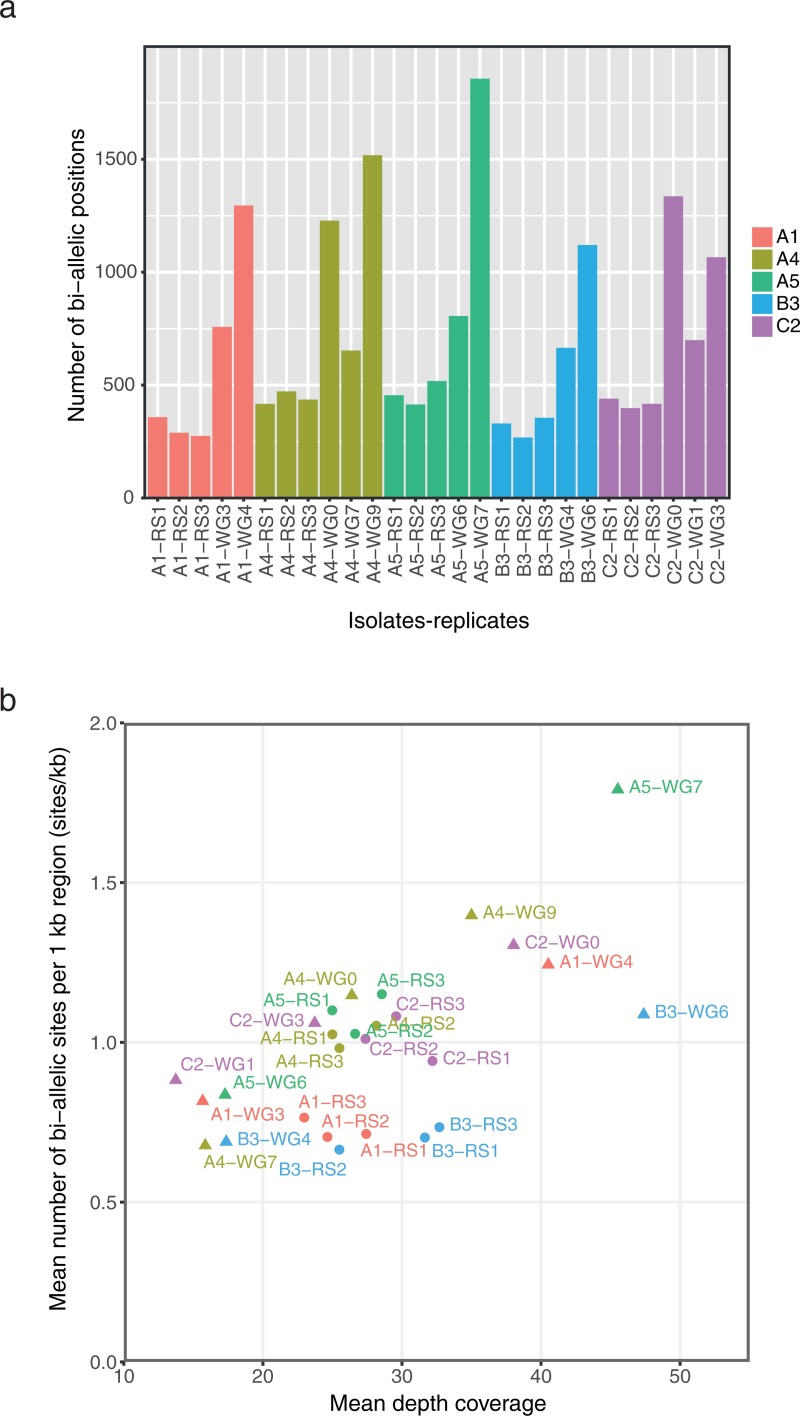
Detection of poly-allelic positions in whole genome (WG) and ddRAD-seq data. (a) Number of bi-allelic positions among replicate samples of the different isolates, where reads were mapped to the corresponding genome. Bi-alleles were analyzed in non-repeated regions, defined with the method M03, and in coding regions. (b) Mean density of poly-allelic positions (composed of SNPs) versus mean depth coverage (excluding ddRAD-seq loci with coverage lower than 10×) in predicted ddRAD-seq regions which are coding and non-repeated (using method 03). "WGx" corresponds to the whole genome sequencing data, where "x" is the replicate identifier. "RSx" corresponds to the ddRAD-seq data, where "x" is the replicate identifier. Replicates of each isolate are shown as separate dots (RS) or triangle (WG).

**Fig 3:**
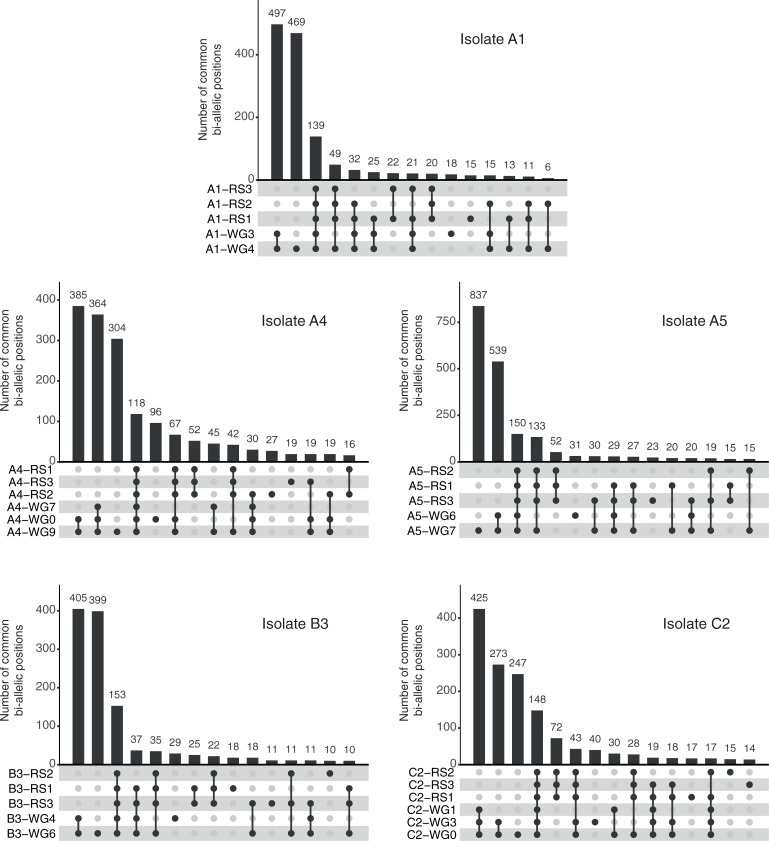
Comparison of bi-allelic sites found in replicates of WG and ddRAD-seq data in five *R*. *irregularis* isolates. Number of replicate samples from WG and ddRAD-seq data that contain the same bi-allelic sites in each isolate. The numbers above the bars in the histograms represent the number of bi-allelic sites shared among a given set of replicates from WG and ddRAD-seq. Bullets connected by vertical lines below the histograms show which replicates contained the same bi−allelic positions. The Up-set plots show numbers of common positions among samples ranked from highest on the left of the graph for the highest 15 combinations of samples. A very small number of bi-allelic sites were found among other samples that are not shown but these numbers are so low as not to alter the conclusions that can be drawn from this analysis.

The mean depth of coverage across the samples ranged from 22 to 33 and from 13 to 47 for ddRAD-seq and WG data, respectively (S4 Fig). A greater number of predicted ddRAD-seq regions in the five isolates were covered by reads from WG data than by ddRAD-seq data (S5 Fig). Poly-allelic positions were almost all bi-allelic (S5 Table). Tri-allelic positions represented approximately 0.5% of the poly-allelic sites. Only one tetra-allelic position was found (S5 Table). Bi-allelic positions were found in all isolates and both types of data (Fig 2A). The density of bi-allelic positions ranged from 0.66 to 1.79 bi-allelic positions/kb (Fig 2B). In general, the density of bi-allelic positions was higher when the depth of coverage was higher (Fig 2B). The majority of bi-allelic sites that were found in the ddRAD-seq datasets also occurred in the WG datasets (Fig 3). In most cases, these occurred in all replicates of the two datasets or in several replicates of the ddRAD-seq data and WG data, confirming that it is very unlikely that they occurred because of random error (Fig 3). Hypergeometric tests showed that the overlap of which fragments from ddRAD-seq and WG mapped to the in silico predicted RAD fragments was highly unlikely to be due to chance (Cumulative hypergeometric test (function *phyper*), P < 0.00001, in all isolates). Alternative approaches to determining non-repeated regions yielded very similar results (S6 and S7 Figs) meaning that the detection of within fungus SNPs was not strongly sensitive to the method of repeat detection. There were many more bi-allelic positions in the WG data that were not detected in the ddRAD-seq data simply because so many more predicted ddRAD-seq regions were covered by reads in WG data than in RD data (see S5 Fig).

### Allele frequency similarities

**Fig 4:**
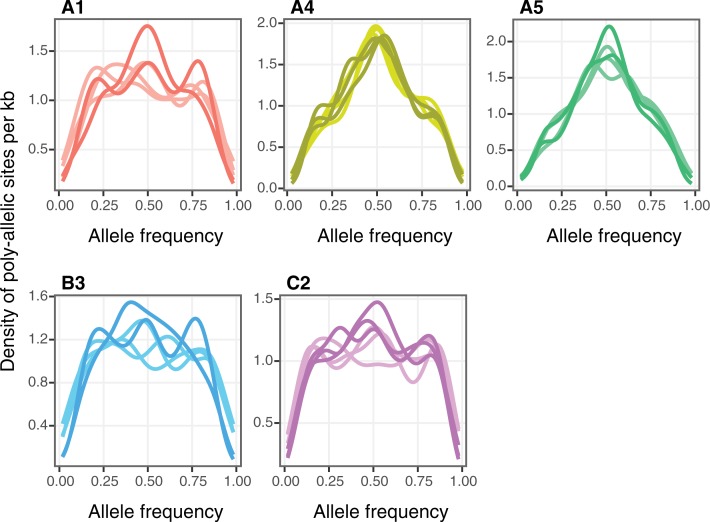
Comparison of distribution of allele frequencies in five *R*. *irregularis* isolates observed in WG and ddRAD-seq data. Distribution of allele frequencies at poly-allelic positions (composed of SNPs) in non-repeated and coding regions (using method 03) of the isolates A1, A4, A5, B3 and C2. Dark coloured lines represent WG data and clear lines ddRAD-seq data.

Assuming nuclei are haploid, allele frequency distribution at bi-allelic sites where the sequence is single copy should reveal the number and proportion of different nuclei. Very similar allele frequency distributions were observed among the replicates of each isolate originating from WG and ddRAD-seq data (Fig 4 and S8 Fig). A peak at 50:50 was detected in A4 and A5 (Fig 2B), as expected for a 50:50 heterokaryon and as shown previously [15]. There was a slight peak at 50:50, as well as slight peaks at approximately 20:80 and 80:20, in A1, B3 and C2 (Fig 4). It is also noticeable that the small peaks at 20:80 and 80:20 also existed in the isolates A4 and A5 despite a clear peak at 50:50. ddRADseq and WG data of isolate DAOM197198 was also subjected to the same analysis and the shape of the curve was highly variable among samples and depending on the genome assembly used for mapping. These results are presented in the S9 Fig.

### Effects of coverage and other parameters

We investigated how different parameters can affect the discovery of bi-allelic positions. All highest-covered samples had a depth of coverage lower than 50× (S4 Fig). In Fig 2B, the density of bi-allelic positions was only reported in non-repeated, coding regions of predicted ddRAD-seq fragments and was higher when the depth of coverage was higher. The number of bi-allelic positions was measured in the whole genome and not only in the predicted ddRAD-seq regions (S10 Fig). The greatest numbers of poly-allelic positions were found in samples having the highest coverage depth. Increasing the depth of coverage by sequencing more deeply lead to higher number of bi-allelic positions and no saturation was detected (S10 Fig).

We applied different thresholds on the depth of coverage in order to evaluate the effects of coverage on the discovery of bi-allelic positions and on patterns of allele frequencies (S11 Fig). Increasing the coverage thresholds had a strong impact on allele frequency distribution. At higher thresholds, the signal around 50:50 disappeared, including the strong 50:50 peaks found for A5 and A4. High thresholds led to a U-shape distribution similar to what was observed by Ropars et al. (2016) [15].

### Bi-allelic positions within RNA transcripts

We used RNA-seq data to explore whether within-isolate polymorphism was detectable in transcripts. We first tested the pipeline on data generated from different species for which the ploidy level is known or for which the states homokaryon, heterokaryon, homozygote and heterozygote is already known. The pipeline clearly demonstrated its efficiency to distinguish heterozygotes from homozygotes, and heretokaryons from homokaryons (S12 Fig and S1 Results).

**Fig 5:**
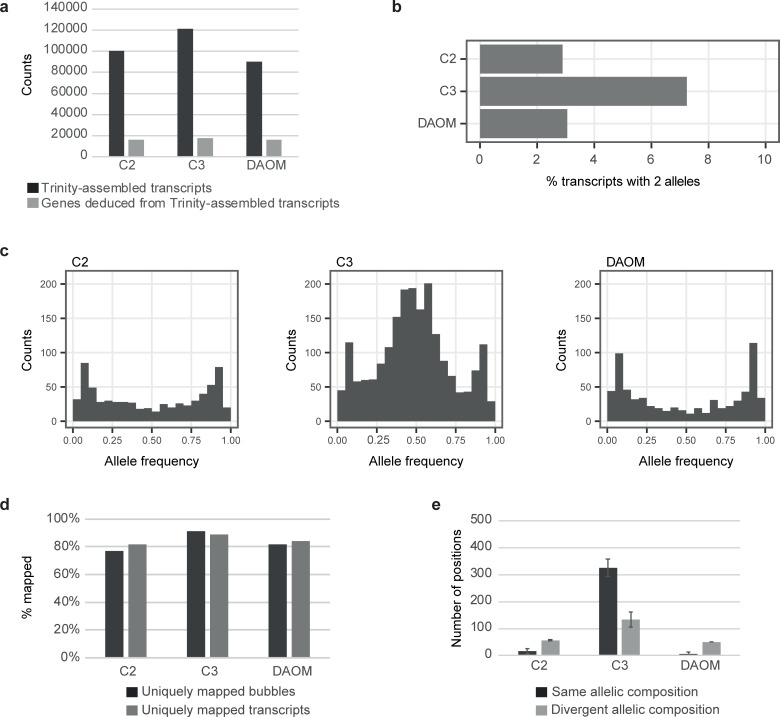
Detection of bi-allelic positions in RNA-seq data. (a) Number of transcripts (black) and genes (grey) obtained with the de-novo transcript assembly approach. (b) Percentage of transcripts containing bi-allelic positions in isolates C2, C3 and DAOM197198. (c) Allele frequency graphs of RNA-seq data at bi-allelic sites. (d) Mapping percentages of transcripts (grey) and bubbles (black) on reference assemblies. Sets of bubbles and transcripts are mapped on the appropriate assembly for each isolate. (e) Number of bi-allelic positions detected in RNA-seq data that have the same allele composition (black) or divergent allele composition (light grey) compared to WG data. Error bars represent ± 1S.E.

The number of transcripts assembled independently from the reference genome was greater in the isolate C3 than in the isolates C2 and DAOM197198 when no filtration was applied (Fig 5A). However, the number of genes deduced from Trinity-assembled transcripts was very similar in the three isolates (Fig 5A). The number of genes recovered in the transcripts represented 60.5%, 68%, and 61.8% of the genes that are thought to be present in the *R*. *irregularis* genomes of C2, C3 and DAOM 197198, respectively. This is based on recent gene predictions of C2, A4 (a clone of C3) and DAOM 197198 [30]. This meant that the RNA-seq datasets from these isolates should have allowed the recovery of a significant proportion of the bi-allelic transcripts, if they exist. Transcripts with bi-allelic positions were found in all isolates (Fig 5B), with a greater proportion in C3 than in C2 and DAOM197198. Detailed results can be found at https://github.com/FredMasc/PAAT2019. The percentage of bi-allelic sites with synonymous codons was 54%, 41% and 37% in C3, C2 and DAOM, respectively.

Allele frequencies in C3 displayed a peak centred at 50:50, although the peak was not as clear as in some of the diploid or heterokaryons shown in S12 Fig. There were two additional peaks at 10 and 90. C2 and DAOM197198 allele frequency plots did not exhibit a 50:50 peak (Fig 5C). U-shaped distributions were observed in C2 and DAOM197198. In addition, moderate peaks were observed at 10:90 and 90:10 in the three isolates. Such patterns are very similar to the patterns observed in the ddRAD-seq and WG data (Fig 4 and S10 Fig).

### Testing for the existence of matching bi-alleles in the *R*. *irregularis* transcriptome and genome

We tested whether bi-alleles found in the transcriptome of the three isolates were also detected independently in the genome. Moderate peaks at 0.1 and 0.9 in the frequency plots (Fig 5C) could be due to wrongly called SNPs because pairs of bubbles do not belong to single transcripts. We tested if all the bubble sequences produced by KisSplice mapped unambiguously to their corresponding reference genome. Both bubble and whole transcript sequences showed similar, and high, proportions of transcripts mapping a single time to the reference genome (Fig 5D). Second, these peaks could be due to sequencing errors. We tested if the positions with two alleles found in RNA-seq data also displayed the same two alleles in WG data. If the bi-alleles are located on two genetically-different nuclei then the same alleles should be found in RNA-seq data and WG data. A total of 326 identical bi-allelic positions occurred in both RNA-seq and WG data in the isolate C3 (Fig 5E). A very small number of bi-allelic positions found in RNA-seq data were also found in WG data in C2 and DAOM197198 (17 in C2 and 5 in DAOM197198). Most of the bi-allelic positions detected in RNA-seq data in C2 and DAOM197198 were not found in WG data (depicted by grey bars and labelled as “divergent allele composition”; Fig 5E). An example of inconsistent positions in C2 is provided in S13 Fig. As a comparison, an example of consistent positions in C3 is shown in S14 Fig. A notably larger number of bi-allelic positions occurred in RNA-seq data that were not found in WG data in isolate C3 compared to C2 and DAOM197198 (Fig 5E).

### Impact of unsolved regions on the within-isolate polymorphism

Each position was visually inspected in the WG read alignment to the reference genome. Bi-allelic positions in C2 and DAOM197198 were frequently found around problematic regions in the genome assembly. Problematic regions were either unsolved regions (gaps) in the genome assembly or regions where the depth of coverage shows an unexpected increase compared to the mean depth of coverage. Both types of problematic regions are frequently found in close locations. Unsolved regions are regions in which Ns are reported in the middle or at the extremities of the scaffold sequences. They correspond to genomic regions that are difficult to assemble. The unsolved regions, which are likely to occur in repeated regions, led to the reads to map wrongly and to accumulate at the wrong locations. This transfer of reads generated bi-allelic positions containing frequently two alleles with equal proportions. Examples of unsolved regions and their effect on the detected within-isolate polymorphism are provided in S15 Fig.

## Discussion

Here we demonstrated that studies of within and among-isolate SNP variation in *R*. *irregularis*, conducted independently, and using different techniques produced remarkably similar results. We have also made the first analysis linking within-AMF isolate variation at the genomic level to within-fungus transcription at bi-allelic sites indicating that in a heterokaryon *R*. *irregularis*, transcription of two alleles at bi-allelic sites is consistent with the proportion of the two genetically different nuclei but that in homokaryons little evidence exists for any expression of possible within fungus genetic variation. The implications of these findings are discussed below.

### Within and among-isolate SNP variation in *R*. *irregularis*

Our analyses indicated that SNP variation within isolates of *R*. *irregularis* were remarkably similar in two studies where the same fungal isolates (or clones of the isolates) were cultured and then sequenced independently in two different laboratories and using two different sequencing approaches. Not only were many of the same SNPs identified in the two studies but they were repeatedly observed in replicates within and between the studies (Fig 3). The same bi-allelic SNPs observed both among replicates and between independent studies means that the probability that they occurred in the sequence datasets by chance is exceptionally unlikely. Furthermore, allele frequency plots among samples from the two studies are also remarkably similar (Fig 4). This analysis shows that for detection of SNPs in *R*. *irregularis*, both approaches are equally valuable for SNP discovery in this fungal species, with the only differences being that more SNPs can be discovered with whole genome sequencing but more isolates can be screened for the same cost using ddRAD-seq. In both cases, sufficient sequencing depth is required and the fact that deeper sequencing reveals more SNPs means that deep sequencing might lead to discovery of a greater number of rare alleles. However, this should also be coupled with replication to ensure rare alleles truly exist.

The discrepancies between the studies are that Ropars et al. (2016) reported lower within-fungus polymorphism than Wyss et al. (2016), although this differed greatly among isolates with values for some isolates being very close and others more distant [13, 15]. One reason is that Wyss et al. (2016) mapped reads to the only available reference genome at that time and because of the very large amount of among-isolate genetic variation. However, the existence of artefacts arising from mapping to one reference genome still does not account for the discovery of consistent among replicate or between-study within-fungus variation observed in each isolate. In the case of isolates considered heterokaryons, these likely represent differences between the two nucleus genotypes or some artefact of mapping where problematic parts of the genome assembly occur. In isolates considered homokaryons, bi-allelic SNPs could either represent artefacts due to problematic parts of the genome assembly or rare alleles.

Allele frequency plots of some isolates reported in Wyss et al. (2016) did not exhibit the U-shaped curves [13]. However, Ropars et al. (2016) observed U-shaped curves in homokaryons but did not explain what accounted for such a distribution [15]. In this study, we show that the shape of the allele frequency distribution is highly sensitive to the read threshold number used as a cut-off (S11 Fig). At a low threshold of 10, almost all isolates appeared to have multiple bi-alleles with many different proportions; even those claimed to be homokaryon. It is difficult to set a standard threshold for all isolates at which homokaryons appear as homokaryons and heterokaryons reveal a 0.5 peak of allele distribution. A higher threshold sets a bias for where only high coverage variants are retained and these usually represent areas of poor assembly or repeated regions. Given that setting a read threshold number is arbitrary, this makes distinction between homokaryons and heterokaryons difficult.

As single cell and single nucleus sequencing technologies improve, a potentially better way to investigate within-fungus genetic variation will likely not have to refer to allele frequencies but hopefully will be able to estimate the true proportions of nuclei carrying a given allele. Such an approach has been successfully used although hopefully the improvement of such technology will allow direct sequencing of larger numbers of individual nuceli [31].

### Bi-allelic variation in the *R*. *irregularis* genome and its transcription

While there has been considerable effort in documenting within and among isolate genome variation in AMF, little attention has been paid whether this variation is transcribed. The number of bi-allelic transcripts that were observed in the isolates expected to be homokaryons were indeed low and only a very small number matched those observed in the genome SNP data (Fig 5E). Of those, most mapped to problematic regions of the genome assembly indicating that these are probably artefacts. A notably large number of alleles in the heterokaryon C3 was observed as 2 alleles in the transcript data and in the genome. We assumed that bi-allelic transcripts that did not have corresponding alleles in the genome data were artefacts (light grey bars in Fig 5E). However, these were notably higher in C3 than in the other two isolates which would not be expected if they occurred because of sequencing errors. Some bi-allelic positions were not discovered in the genome of this isolate because the coverage of genome data is insufficient. The results show that indeed, genes of the two different nucleus genotypes present in the heterokaryon C3 are transcribed. About 50% of these bi-alleles in C3 contained non-synonymous substitutions meaning that this variation among nuclei could potentially affect the fungal phenotype. The allele frequency graphs of bi-allelic SNPs in the genome and those observed independently in the transcriptome both revealed an allele frequency of 0.5. This suggests that in isolate C3, the two nucleus genotypes are present in roughly equal proportions; a measurement that was also corroborated by a separate study [32] using the allele frequencies of a single marker and replicate measurements on the same isolate. The 0.5 allele frequency in the transcripts suggests that the expression level from a pair of given alleles located on the two different nuclei is similar In several studies from our group, sibling cultures of AMF, initiated from single sibling spores for the parent C3 have been shown to exhibit quantitative traits such as hyphal growth and spore production, as well as their effects on plant growth, that were significantly different from the parent C3 [4, 5]. This is highly unusual for clonal offspring. Recently, the ratios of the two nucleus genotypes in some of these sibling offspring lines were shown to deviate significantly from the 50:50 ratio observed in the parent C3 [32]. If gene transcription from the two nucleus genotypes is proportional to the frequency of those genotypes (as shown in the present study) then we could expect that sibling clonal offspring of C3 could have unequal transcription of bi-alleles and this could account for the differences in quantitative traits observed among single spore clonally produced siblings of C3. In other, fungi such as *Agaricus bisporus*, nucleus specific expression has shown that different nuclei exhibit different regulatory programs and this would be an interesting future avenue to pursue in this AMF species [33].

## Conclusions

Investigating whether within-AMF isolate genetic variation is extremely difficult. Independent studies using different techniques have identified the same variants and we show that this has obviously not occurred by chance. Transcription profiling is a good way to verify some of these bi-allelic positions in the genome. Transcription profiling did not reveal significant transcription of bi-allelic sites in a homokaryon isolate. However, transcription profiling reveals that indeed, within fungus genetic variation existing in heterokaryons is transcribed from both of the co-existing genomes and in equal proportions from both nucleus genotypes. Further studies are needed to determine the nature of the genetic differences between these two genotypes and how their expression influences the fungal phenotype and its effects on plants.

## Supporting information

S1 Results This file contains supplementary results on testing the Ksplice pipeline on RNA-seq data generated from different species as well as supplementary methods. (DOCX)

S1 Fig Methodology to detect poly-allelic positions in whole genome (WG) and ddRAD-seq (RS) data. Schematic diagram of the methodology used to discover potential within-fungus genetic poly- morphism. Line 'G' corresponds to a linear part of a genome where EcoRI and MseI restriction sites are marked. Line 'P' shows a black bar corresponding to a predicted RAD fragment between EcoRI and MseI restriction sites. Line 'R' shows a green bar corresponding to a repeated region in the genome. Line 'C' shows a violet bar corresponding to a coding region in the genome. Lines 'WG' and 'RS' show respectively whole genome sequencing reads and ddRAD-seq reads mapped to this genomic region. Line 'P-A' shows the position where poly-allelic positions are found and the alleles found at this position. A semi-transparent orange box delimits the region of interest: in the predicted RAD fragment, in coding region and not in the repeated region. The example shows a position where alleles T and C can be found. In the empirical data, most poly-allelic positions are bi-allelic positions.
(PDF)

S2 Fig Analysis pipeline of RNA-seq data. (a) Overview of the pipeline including KisSplice to call SNPs from RNA-seq data without a reference genome. (b) Example of a ‘bubble’ structure shaped in a De Bruijn graph at a given bi-allelic position.
(PDF)

S3 Fig Effects of the reference genome on the density of bi-allelic positions. Each line represents the density of bi-allelic positions detected in one replicate when ddRAD-seq reads were mapped to different reference genomes. A specific colour is used for each isolate. Bi-alleles were analyzed in non-repeated regions, defined with the method M12, and in non-coding regions.
(PDF)

S4 Fig Boxplot of the depth of coverage of ddRad-seq (RS) data and whole genome sequencing (WG) data. All samples were aligned to their respective genomes. The boxplot depicts the distribution of depth of coverage of all ddRAD-seq and whole genome sequencing samples. Artifactual sequences present in the ddRAD-seq data were eliminated by applying a minimal coverage threshold of 10×.
(PDF)

S5 Fig Percentage of predicted ddRAD-seq regions covered with ddRAD-seq reads and whole genome sequencing (WG) reads. Blue bars correspond to ddRAD-seq data and red bars correspond to WG data. Only ddRAD-seq regions with a depth coverage threshold of 10× were recorded. Graphs were gene- rated in non-repeated and coding ddRAD-seq regions, following the M03 method to identify the repeats.
(PDF)

S6 Fig Number of common bi-allelic positions in non-repeated (defined with method 07) and coding genomic regions among samples in different isolates of *R*. *irregularis*. (a) Number of bi−allelic positions among replicate samples of different isolates. (b), (c), (d), (e) and (f) Number of common bi−allelic positions among samples of A1, A4, A5, B3 and C2 respectively. Numbers of common bi−allelic positions are shown with UpSet plots. The verti- cal lines connecting bullets show common bi−allelic positions in each set of samples.
(PDF)

S7 Fig Number of common bi-allelic positions in non-repeated (defined with method 12) and coding genomic regions among samples in different isolates of *R*. *irregularis*. (a) Number of bi−allelic positions among replicate samples of different isolates. (b), (c), (d), (e) and (f) Number of common bi−allelic positions among samples of A1, A4, A5, B3 and C2 respectively. Numbers of common bi−allelic positions are shown with UpSet plots. The verti- cal lines connecting bullets show common bi−allelic positions in each set of samples.
(PDF)

S8 Fig Scatterplots showing the relationship between allele frequency at bi-allelic sites observed in whole genome sequencing data and ddRADseq data from five isolates of *R*. *irregularis* (A1, C3, A5, B3 & C2). (PDF)

S9 Fig Analysis of frequencies at bi-allelic sites in isolate DAOM197198. (a) Allele frequency plots when assembly DNA1 was used as a reference genome (only coding and non-repeated regions). (b) Allele frequency plots when assembly DNA2 was used as a reference genome (only coding and non-repeated regions). (c) Boxplot of depth of coverage for ddRAD-seq data and WG data of the isolate DAOM197198. (d) Mean number of bi-allelic positions per 1 kb region *versus* depth of coverage detected in WG data in non-repeated and non-coding regions. This figure is the same as Fig 2B with the addition of DAOM197198 ddRAD-seq and WG samples (pink).
(PDF)

S10 Fig Mean number of bi-allelic positions versus mean depth of coverage detected in whole genome (WG) data in non-repeated and coding regions across the whole genome assembly. The whole genome assembly was considered and not only the predicted ddRAD-seq regions.
(PDF)

S11 Fig Allele frequency graphs obtained with different depth coverage thresholds. All positions in the genome, except the regions labelled as ‘repeats’, were considered for this analysis. Ran- domly chosen frequencies of one allele at di-allelic positions were considered in order to generate the distri- bution. Only positions with a total depth coverage higher or equal to the threshold were included.
(PDF)

S12 Fig Analysis of RNAseq data in other fungi and plants. (PDF)

S13 Fig Examples of bi-allelic positions found in RNA-seq data and not in WG data in isolate C2. (a), (b) and (c) correspond to three examples in different regions of the genome displayed in IGV browser. Only nucleotides differing from the reference genome are shown with a colour different from light gray. Alleles contributing to bi-allelic positions in RNA-seq are surrounded with red circles or ellipses.
(PDF)

S14 Fig Examples of bi-allelic positions found both in RNA-seq data and in WG data in isolate C3. (a), (b) and (c) correspond to three examples in different regions of the genome displayed in IGV browser. Only nucleotides differing from the reference genome are shown with a colour different from light gray. Alleles contributing to bi-allelic positions in RNA-seq are surrounded with red circles or ellipses.
(PDF)

S15 Fig Examples of problematic regions in the genome assembly of the isolate C2. (a), (b) and (c) correspond to three examples in different regions of the genome displayed in IGV browser. Colours other than light grey depict alleles different from the reference in the reads. ‘Depth’ shows a histogram of the depth of coverage for genomic reads. Interesting regions are labelled with red text and boxes.
(PDF)

S1 Table Sources of whole genome sequencing data. (XLSX)

S2 Table Sources of ddRAD-seq data. (XLSX)

S3 Table Sources of RNA-seq data. (XLSX)

S4 Table Read length after quality control treatment. (XLSX)

S5 Table Counts of bi- tri- and tetra-allelic positions. (XLSX)
