# Supplementary material for: Investigating unexplained genetic variation and its expression in the arbuscular mycorrhizal fungus Rhizophagus irregularis: A comparison of whole genome and RAD sequencing data
Source: PLoS One. 2019 Dec 27;14(12):e0226497. doi: 10.1371/journal.pone.0226497 (PMC6934306; doi:10.1371/journal.pone.0226497)
Supplement: S1 Results — (DOCX) [file pone.0226497.s001.docx]

**Supplementary results**

**Test of the pipeline including KisSplice on RNA-seq data generated from different species**

We investigated the level of within-isolate polymorphism in transcripts of two homokaryote and one dikaryote isolate of the fungus *Antrodia cinnamomea*. More transcripts with poly-allelic positions were found in the dikaryote than in the homokaryotes (Figure S12a). The allele frequency graphs showed a peak at an allele ratio of 50:50 in the dikaryon (Figure S12a). It is also the typical peak expected for a dikaryon having an equal ratio of different nuclei that equally express transcripts. Only a weak random signal, considered as artefactual, was observed in the allele frequency graphs of the homokaryons (Figure S12a). The dikaryotes could be unambiguously identified among the homokaryotes and the allelic transcripts are expressed, on average, at the same level.

The pipeline was also tested with four *Arabidopsis* RNA-seq samples. Three of the samples were natural diploid accessions and mostly homozygous as *Arabidopsis* is self-fertilizing. The fourth sample was a lab-generated F1 hybrid between two different natural accessions and can be considered as a strong heterozygote. Analysis of the allele frequency graphs clearly showed an artefactual signal for the three natural accessions and a high 50:50 peak for the hybrid (Figure S12b). The heterozygote could be unambiguously identified among the homozygotes and the allelic transcripts are expressed on average at the same level.

RNA-seq data from other plants with different levels of ploidy were also subjected to the pipeline. *Aegilops longissimi* is a diploid homozygote that was hybridized with another diploid homozygote (*Triticum urartu*) to create the heterozygote tetraploid hybrid. Only an artefactual signal was found for the homozygote *A. longissimi*, while a high 50:50 peak was found for the heterozygote tetraploid hybrid plant (Figure S12c). The heterozygote could be unambiguously distinguished from the homozygote and the transcripts of bi-alleles were expressed, on average, at the same level.

Analysis of RNA-seq data from the haploid *Saccharomyces cerevisiae*, revealed no obvious peak, as expected. The heterokaryote *Rhizoctonia solani* exhibited a 50:50 allele frequency peak, although some isolates of this species have been reported to be heterokaryotic and harbouring more than 2 genetically different nuclei. If this was the case in this study then the pipeline was not able to resolve more complex within-fungus genetic structure. The diploid *Candida albicans* exhibited an expected 50:50 peak (Figure S12d). Allelic transcripts in the heterokaryote and in the diploid were expressed, on average, in similar proportions.

In all the tested RNA-seq data from different species the pipeline, including KisSplice, demonstrated its efficiency to distinguish the heterozygotes (and heterokaryotes) from the homozygotes (and homokaryotes) without ambiguity.

**Supplementary material and methods**

**Versions, flags and options used for bioinformatics programs**

**Genomics**

tagcleaner.pl version 0.14

-tag5 CCTACACGACGCTCTTCCGATCT (forward reads)

-tag3 AGATCGGAAGAGCACACGTCTGA

-matrix exact

-trim_within 60

-mm5 3

-mm3 4

-tag5 TCAGACGTGTGCTCTTCCGATCT (reverse reads)

-tag3 AGATCGGAAGAGCGTCGTGTAGG

prinSeq-lite.pl version 0.20.4

-trim_ns_left 1 -trim_qual_type "mean" -trim_qual_window 10 -trim_qual_step 9 -trim_qual_right 15 -min_len 50

process_radtags version 1.06 (STACKS)

-E phred33 -r -e ecoRI -c -q -D -s 10

Novoalign 3.02.12

Standard parameters

samtools version 1.3

Standard parameters

freebayes version 1.0.2

-p 10 -J -K -F 0.1 -0 -u

vcffilter 1.0

-f 'QUAL > 30'

RepeatModeler Open-1.0

Standard parameters

RepeatMasker Open-3.0

Standard parameters

fasta-36.3.5e

Standard parameters

**Transcriptomics**

Assemblies with Trinity

Trinity version 2.5.1

bowtie2 version 2.3.0

java version 1.8.0_121

Trinity

--trimmomatic --max_memory 100G --CPU 60 --SS_lib_type RF --full_cleanup

kissplice version 2.4.0

--experimental --max-memory 6000 -v -s 2 -k 41 -t 10

ncbi-blast version 2.6.0+

Standard parameters

TransDecoder-v5.0.2

Standard parameters

Blat v36

-minIdentity=80

**Structural variations**

Speedseq v0.1.2

Standard parameters (as specified in LUMPY manual)

lumpyexpress (LUMPY 0.2.13)

Standard parameters

SVTyper 0.7.0

Standard parameters

CNVnator 0.3.1

Standard parameters

Subsequent filter for scaffold size (10000), p-value n°1 (< 0.05), q0 value (< 0.5)

**Custom Perl and R scripts**

Custom Perl and R scripts used to treat the data in Genomics, Transcriptomics and Structural variations are availalble at https://github.com/FredMasc/PAAT2019
