# Supplementary material for: Investigating unexplained genetic variation and its expression in the arbuscular mycorrhizal fungus Rhizophagus irregularis: A comparison of whole genome and RAD sequencing data
Source: PLoS One. 2019 Dec 27;14(12):e0226497. doi: 10.1371/journal.pone.0226497 (PMC6934306; doi:10.1371/journal.pone.0226497)
Supplement: S1 Fig — Schematic diagram of the methodology used to discover potential within-fungus genetic poly- morphism. Line 'G' corresponds to a linear part of a genome where EcoRI and MseI restriction sites are marked. Line 'P' shows a black bar corresponding to a predicted RAD fragment between EcoRI and MseI restriction sites. Line 'R' shows a green bar corresponding to a repeated region in the genome. Line 'C' shows a violet bar corresponding to a coding region in the genome. Lines 'WG' and 'RS' show respectively whole genome sequencing reads and ddRAD-seq reads mapped to this genomic region. Line 'P-A' shows the position where poly-allelic positions are found and the alleles found at this position. A semi-transparent orange box delimits the region of interest: in the predicted RAD fragment, in coding region and not in the repeated region. The example shows a position where alleles T and C can be found. In the empirical data, most poly-allelic positions are bi-allelic positions. (PDF) [file pone.0226497.s002.pdf]

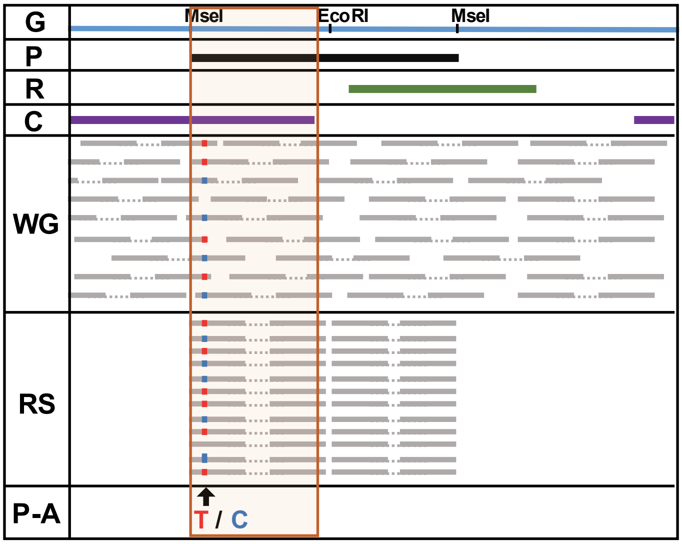

Figure S1. Methodology to detect poly-allelic positions in whole genome (WG) and ddRAD-seq (RS) data. Schematic diagram of the methodology used to discover potential within-fungus genetic polymorphism. Line 'G' corresponds to a linear part of a genome where EcoRI and MseI restriction sites are marked. Line 'P' shows a black bar corresponding to a predicted RAD fragment between EcoRI and MseI restriction sites. Line 'R' shows a green bar corresponding to a repeated region in the genome. Line 'C' shows a violet bar corresponding to a coding region in the genome. Lines 'WG' and 'RS' show respectively whole genome sequencing reads and ddRAD-seq reads mapped to this genomic region. Line 'P-A' shows the position where poly-allelic positions are found and the alleles found at this position. A semi-transparent orange box delimits the region of interest: in the predicted RAD fragment, in coding region and not in the repeated region. The example shows a position where alleles T and C can be found. In the empirical data, most poly-allelic positions are bi-allelic positions.
