# Supplementary material for: Investigating unexplained genetic variation and its expression in the arbuscular mycorrhizal fungus Rhizophagus irregularis: A comparison of whole genome and RAD sequencing data
Source: PLoS One. 2019 Dec 27;14(12):e0226497. doi: 10.1371/journal.pone.0226497 (PMC6934306; doi:10.1371/journal.pone.0226497)
Supplement: S2 Fig — (a) Overview of the pipeline including KisSplice to call SNPs from RNA-seq data without a reference genome. (b) Example of a ‘bubble’ structure shaped in a De Bruijn graph at a given bi-allelic position. (PDF) [file pone.0226497.s003.pdf]

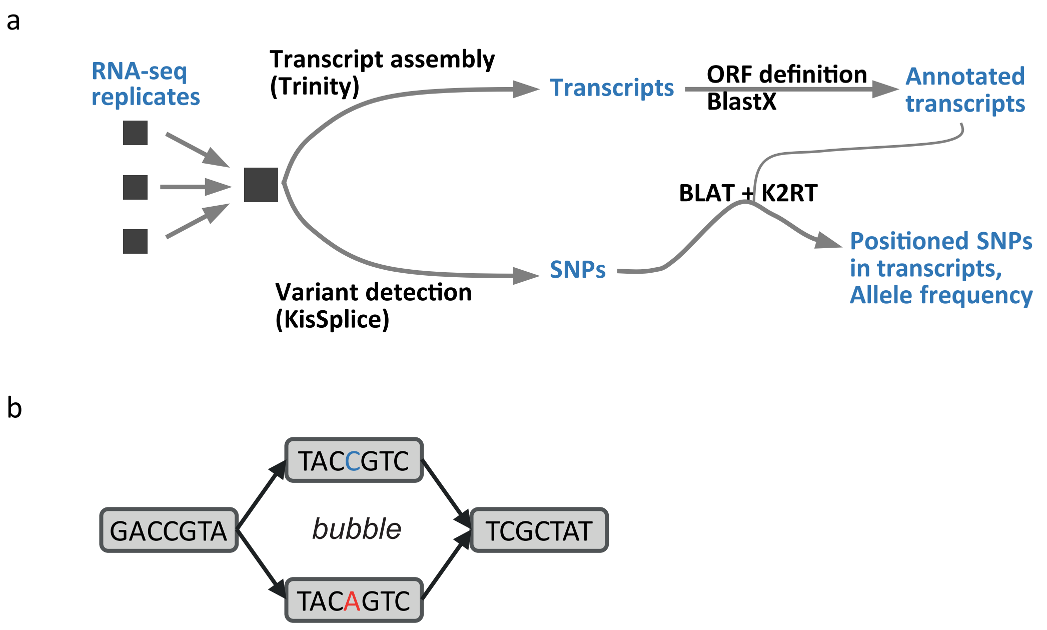

Figure S2. Analysis pipeline of RNA-seq data. (a) Overview of the pipeline including KisSplice to call SNPs from RNA-seq data without a reference genome. (b) Example of a 'bubble' structure shaped in a De Bruijn graph at a given bi-allelic position.
