# Supplementary material for: Investigating unexplained genetic variation and its expression in the arbuscular mycorrhizal fungus Rhizophagus irregularis: A comparison of whole genome and RAD sequencing data
Source: PLoS One. 2019 Dec 27;14(12):e0226497. doi: 10.1371/journal.pone.0226497 (PMC6934306; doi:10.1371/journal.pone.0226497)
Supplement: S3 Fig — Each line represents the density of bi-allelic positions detected in one replicate when ddRAD-seq reads were mapped to different reference genomes. A specific colour is used for each isolate. Bi-alleles were analyzed in non-repeated regions, defined with the method M12, and in non-coding regions. (PDF) [file pone.0226497.s004.pdf]

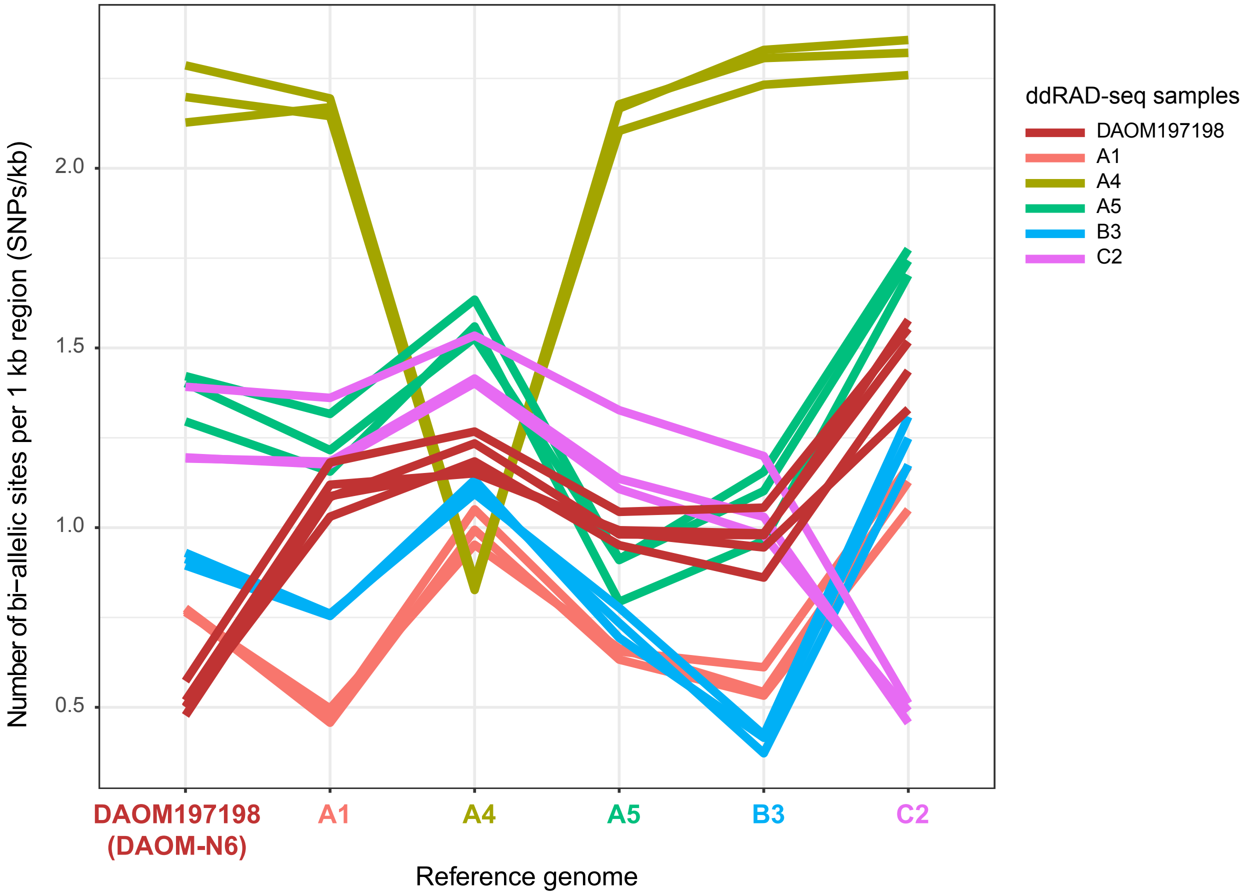

Figure S3: Effects of the reference genome on the density of bi-allelic positions.
Each line represents the density of bi-allelic positions detected in one replicate when ddRAD-seq reads were mapped to different reference genomes. A specific colour is used for each isolate. Bi-alleles were analyzed in non-repeated regions, defined with the method M12, and in non-coding regions.
