# Supplementary material for: Investigating unexplained genetic variation and its expression in the arbuscular mycorrhizal fungus Rhizophagus irregularis: A comparison of whole genome and RAD sequencing data
Source: PLoS One. 2019 Dec 27;14(12):e0226497. doi: 10.1371/journal.pone.0226497 (PMC6934306; doi:10.1371/journal.pone.0226497)
Supplement: S4 Fig — All samples were aligned to their respective genomes. The boxplot depicts the distribution of depth of coverage of all ddRAD-seq and whole genome sequencing samples. Artifactual sequences present in the ddRAD-seq data were eliminated by applying a minimal coverage threshold of 10×. (PDF) [file pone.0226497.s005.pdf]

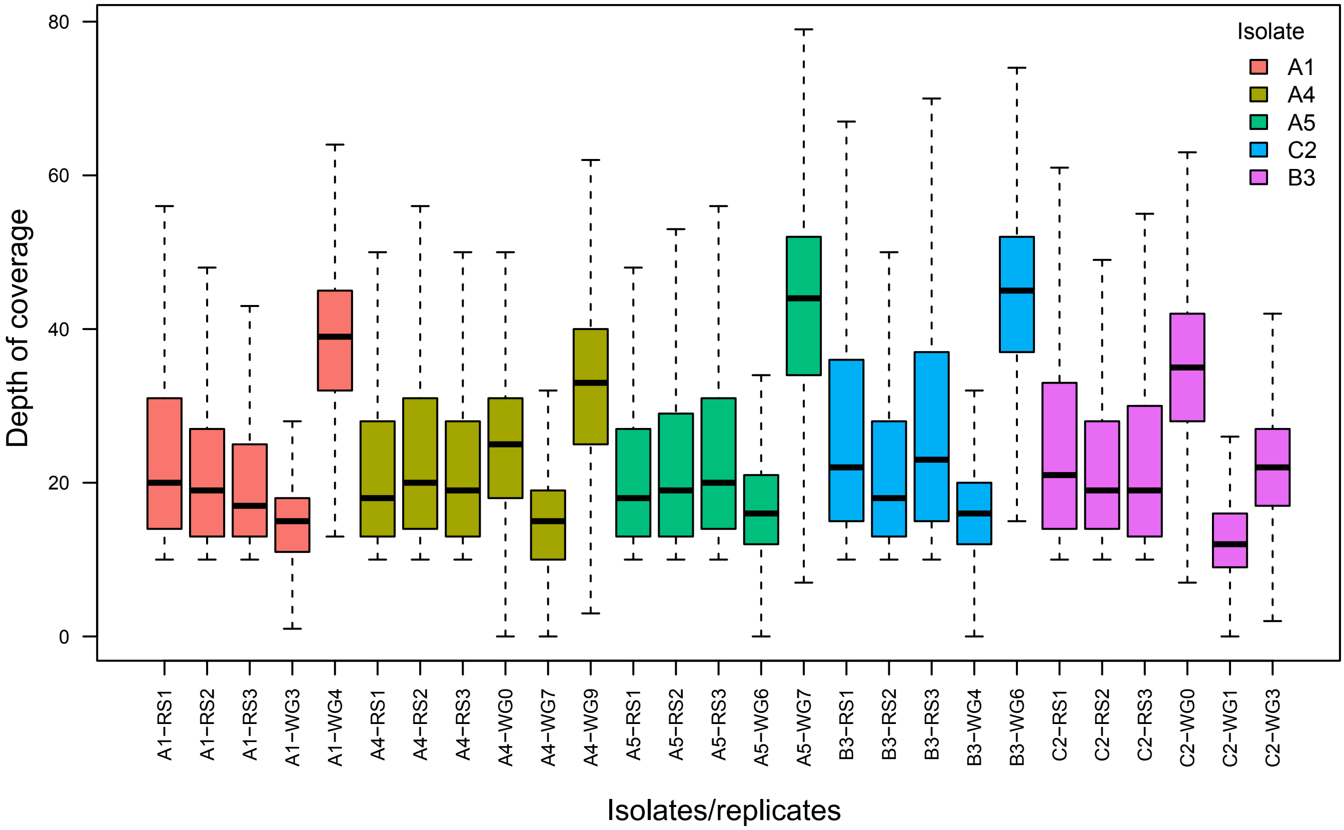

Figure S4: Boxplot of the depth of coverage of ddRad-seq (RS) data and whole genome sequencing (WG) data. All samples were aligned to their respective genomes. The boxplot depicts the distribution of depth of coverage of all ddRAD-seq and whole-genome sequencing samples. Artifactual sequences present in the ddRAD-seq data were eliminated by applying a minimal coverage threshold of 10×.
