# Supplementary material for: Investigating unexplained genetic variation and its expression in the arbuscular mycorrhizal fungus Rhizophagus irregularis: A comparison of whole genome and RAD sequencing data
Source: PLoS One. 2019 Dec 27;14(12):e0226497. doi: 10.1371/journal.pone.0226497 (PMC6934306; doi:10.1371/journal.pone.0226497)
Supplement: S5 Fig — Blue bars correspond to ddRAD-seq data and red bars correspond to WG data. Only ddRAD-seq regions with a depth coverage threshold of 10× were recorded. Graphs were gene- rated in non-repeated and coding ddRAD-seq regions, following the M03 method to identify the repeats. (PDF) [file pone.0226497.s006.pdf]

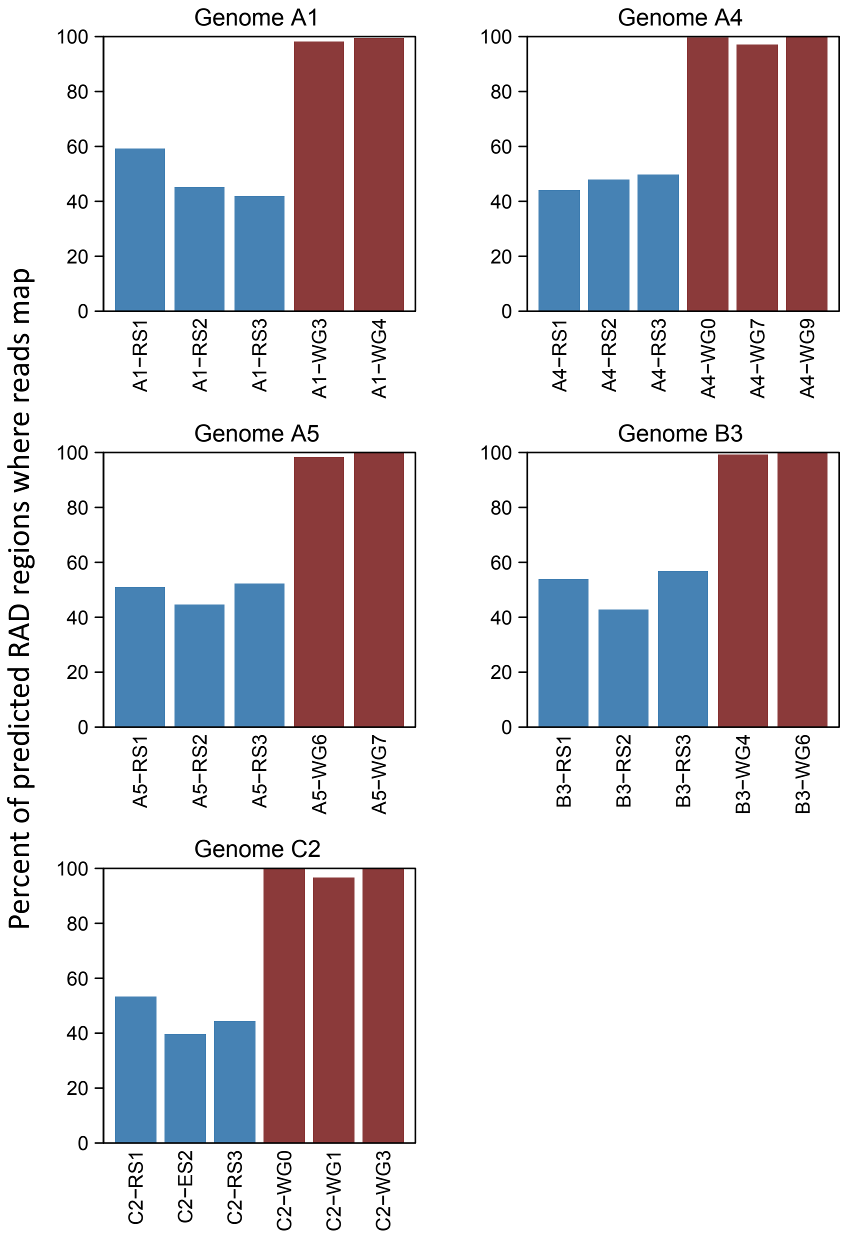

Figure S5: Percentage of predicted ddRAD-seq regions covered with ddRAD-seq reads and whole genome sequencing (WG) reads. Blue bars correspond to ddRAD-seq data and red bars correspond to WG data. Only ddRAD-seq regions with a depth coverage threshold of 10× were recorded. Graphs were generated in non-repeated and coding ddRAD-seq regions, following the M03 method to identify the repeats.
