# Supplementary material for: Investigating unexplained genetic variation and its expression in the arbuscular mycorrhizal fungus Rhizophagus irregularis: A comparison of whole genome and RAD sequencing data
Source: PLoS One. 2019 Dec 27;14(12):e0226497. doi: 10.1371/journal.pone.0226497 (PMC6934306; doi:10.1371/journal.pone.0226497)
Supplement: S7 Fig — (a) Number of bi−allelic positions among replicate samples of different isolates. (b), (c), (d), (e) and (f) Number of common bi−allelic positions among samples of A1, A4, A5, B3 and C2 respectively. Numbers of common bi−allelic positions are shown with UpSet plots. The verti- cal lines connecting bullets show common bi−allelic positions in each set of samples. (PDF) [file pone.0226497.s008.pdf]

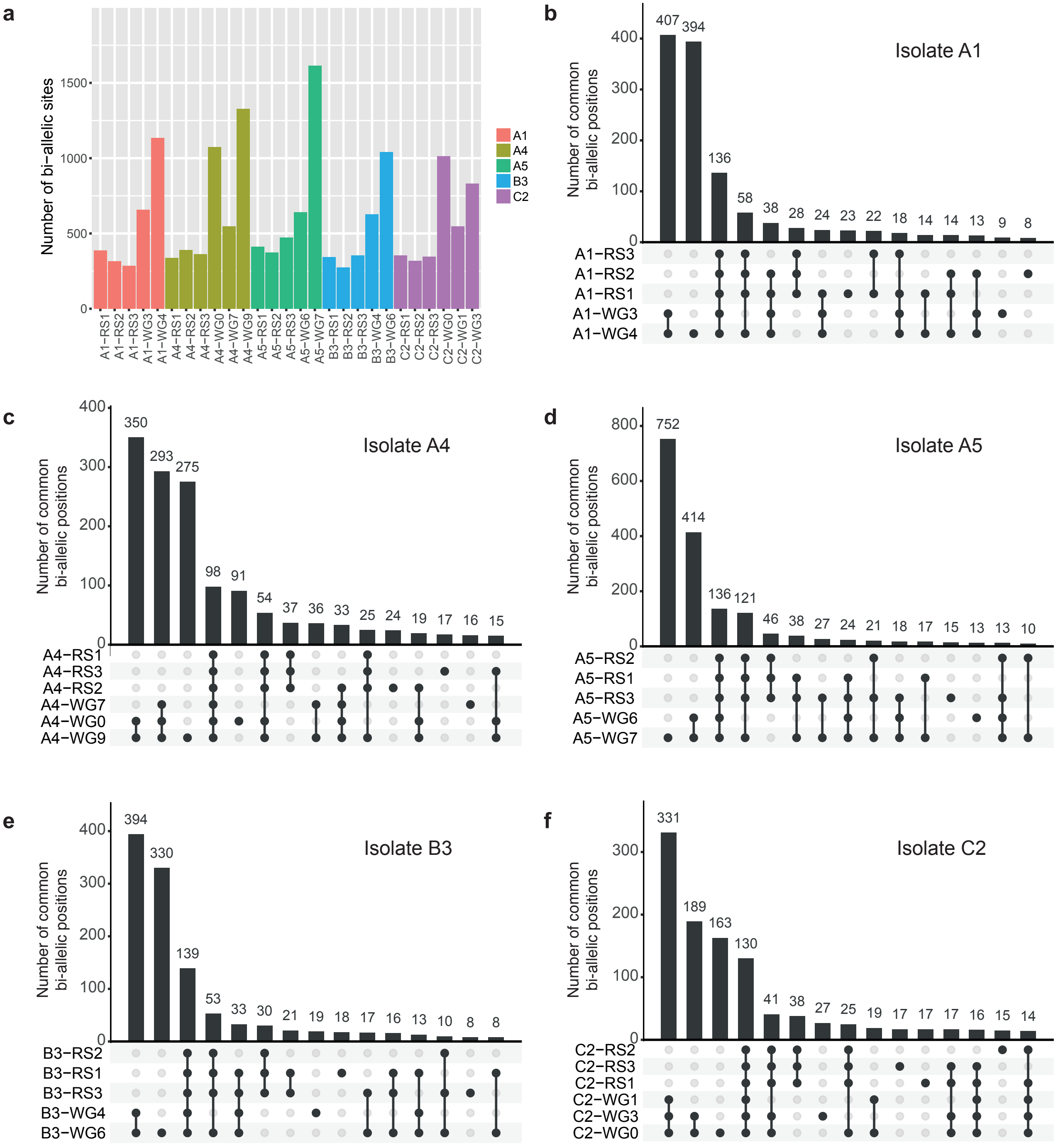

Figure S7: Number of common bi-allelic positions in non-repeated (defined with method 12) and coding genomic regions among samples in different isolates of *R. irregularis*. (a) Number of bi−allelic positions among replicate samples of different isolates. (b), (c), (d), (e) and (f) Number of common bi−allelic positions among samples of A1, A4, A5, B3 and C2 respectively. Numbers of common bi−allelic positions are shown with UpSet plots. The vertical lines connecting bullets show common bi−allelic positions in each set of samples.
