# Supplementary material for: Investigating unexplained genetic variation and its expression in the arbuscular mycorrhizal fungus Rhizophagus irregularis: A comparison of whole genome and RAD sequencing data
Source: PLoS One. 2019 Dec 27;14(12):e0226497. doi: 10.1371/journal.pone.0226497 (PMC6934306; doi:10.1371/journal.pone.0226497)
Supplement: S8 Fig — (PDF) [file pone.0226497.s009.pdf]

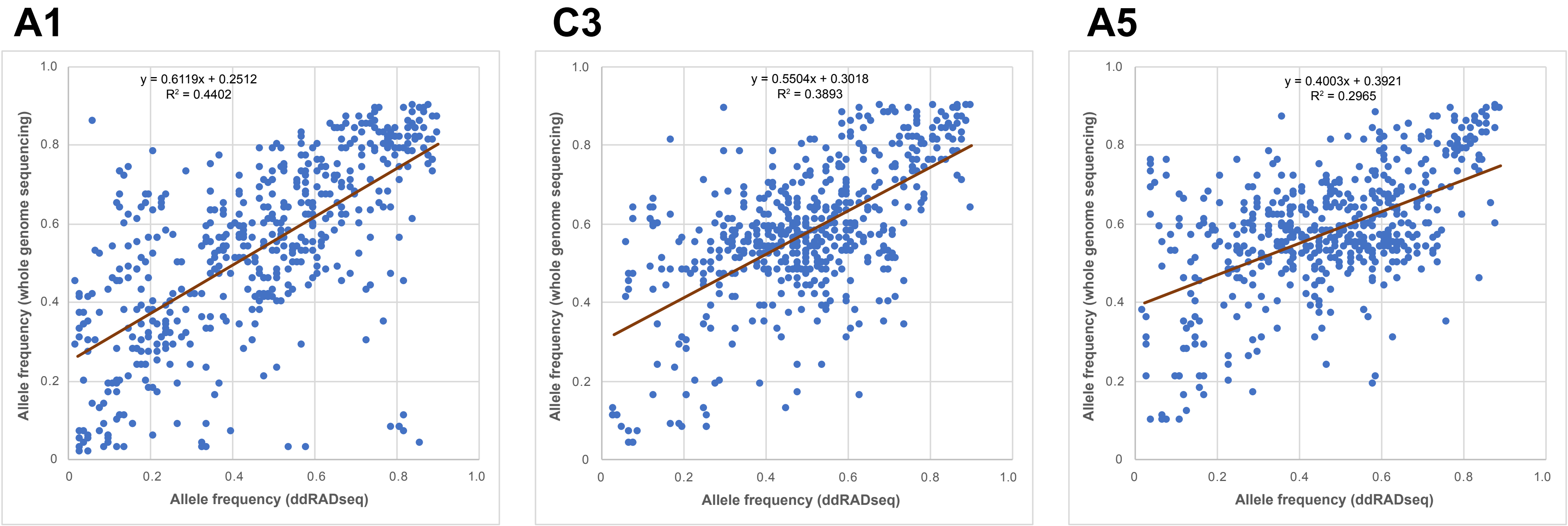

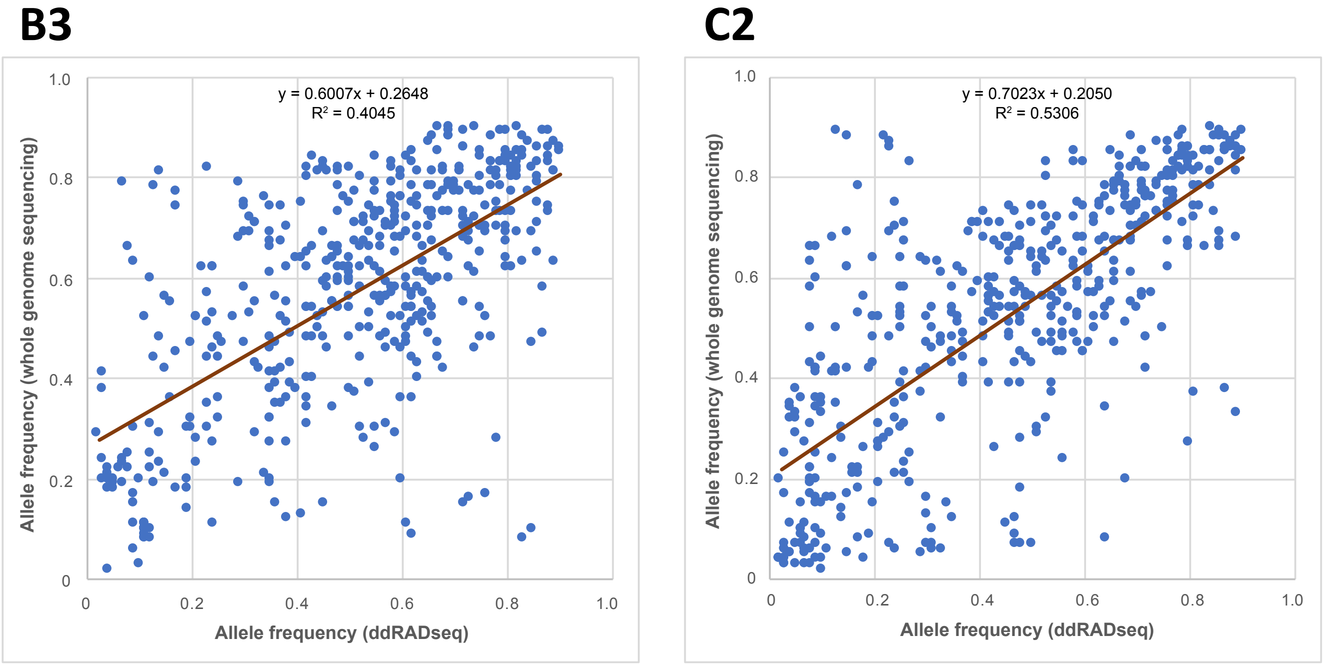

Figure S8. Scatterplots showing the relationship between allele frequency at bi-allelic sites observed in whole genome sequencing data and ddRADseq data from five isolates of *R. irregularis* (A1, C3, A5, B3 & C2).
