# Supplementary material for: Investigating unexplained genetic variation and its expression in the arbuscular mycorrhizal fungus Rhizophagus irregularis: A comparison of whole genome and RAD sequencing data
Source: PLoS One. 2019 Dec 27;14(12):e0226497. doi: 10.1371/journal.pone.0226497 (PMC6934306; doi:10.1371/journal.pone.0226497)

Figure S9. Analysis of frequencies at bi-allelic sites in isolate DAOM197198.

a Allele frequency plots when assembly DNA1 was used as a reference genome
(only coding and non repeated regions)

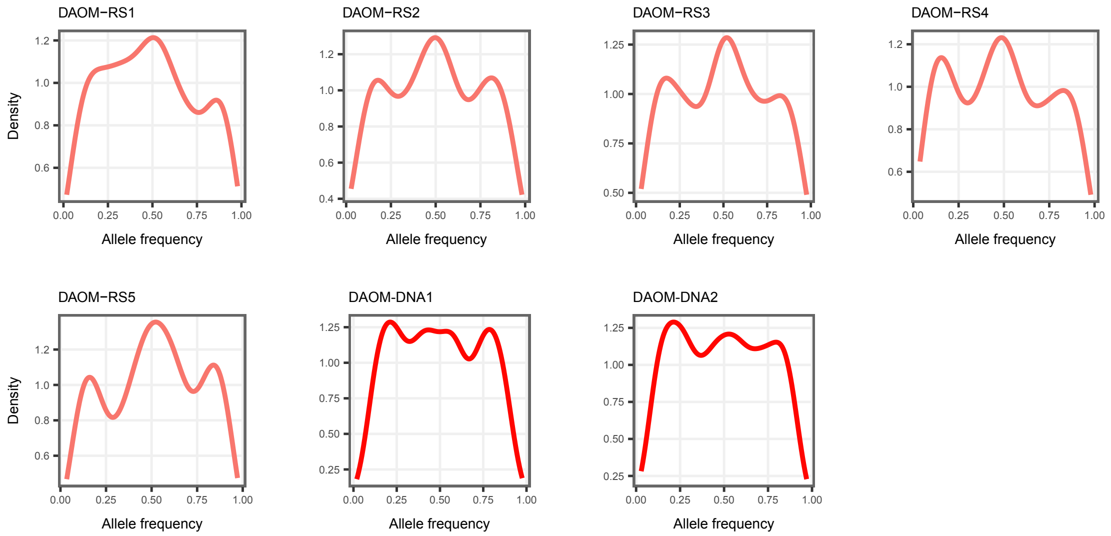

b Allele frequency plots when assembly DNA2 was used as a reference genome
(only coding and non repeated regions)

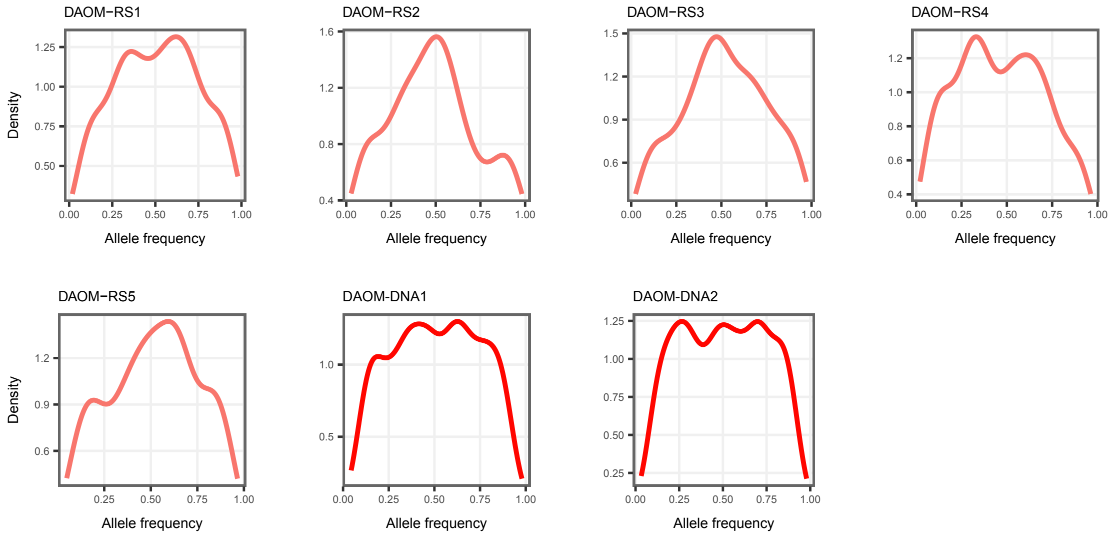

c Boxplot of depth of coverage for ddRAD-seq data and WG data of the isolate DAOM197198.

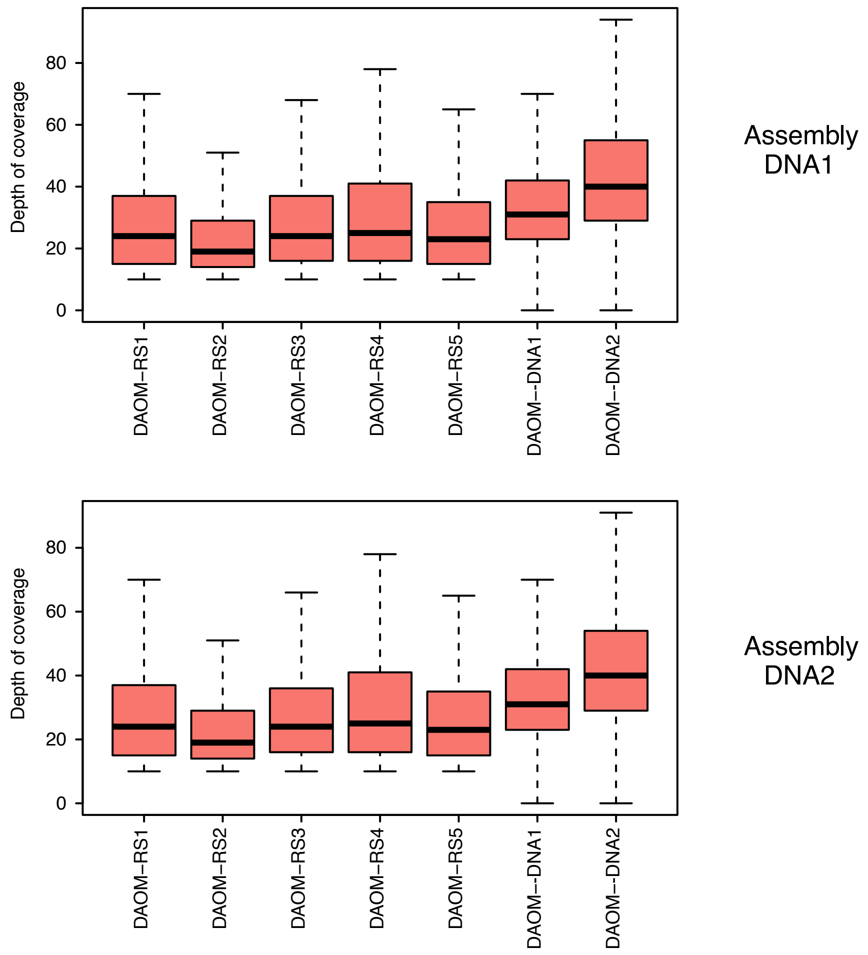

d Mean number of bi-allelic positions per 1 kb region versus depth of coverage detected in WG data in non-repeated and non-coding regions. This figure is the same as figure 2b with the addition of DAOM197198 ddRAD-seq and WG samples (pink).

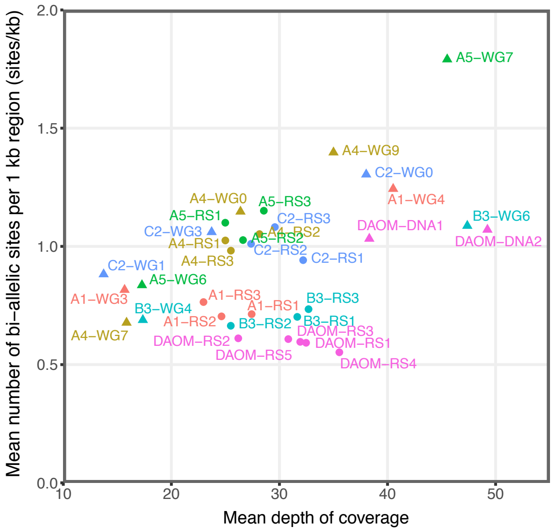

Supplement: S9 Fig — (a) Allele frequency plots when assembly DNA1 was used as a reference genome (only coding and non-repeated regions). (b) Allele frequency plots when assembly DNA2 was used as a reference genome (only coding and non-repeated regions). (c) Boxplot of depth of coverage for ddRAD-seq data and WG data of the isolate DAOM197198. (d) Mean number of bi-allelic positions per 1 kb region versus depth of coverage detected in WG data in non-repeated and non-coding regions. This figure is the same as Fig 2B with the addition of DAOM197198 ddRAD-seq and WG samples (pink). (PDF) [file pone.0226497.s010.pdf]
