# Supplementary material for: Investigating unexplained genetic variation and its expression in the arbuscular mycorrhizal fungus Rhizophagus irregularis: A comparison of whole genome and RAD sequencing data
Source: PLoS One. 2019 Dec 27;14(12):e0226497. doi: 10.1371/journal.pone.0226497 (PMC6934306; doi:10.1371/journal.pone.0226497)
Supplement: S10 Fig — The whole genome assembly was considered and not only the predicted ddRAD-seq regions. (PDF) [file pone.0226497.s011.pdf]

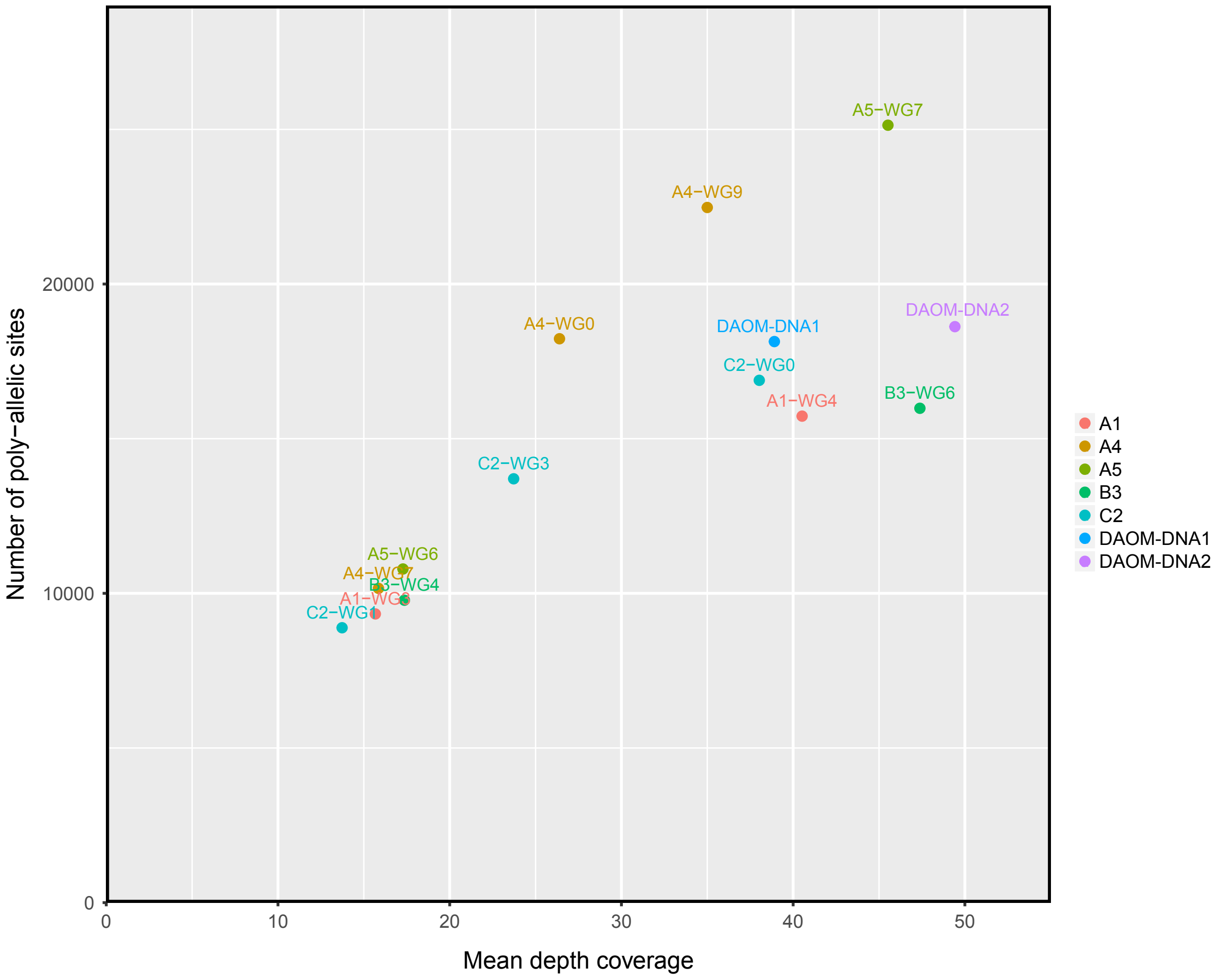

Figure S10: Mean number of bi-allelic positions versus mean depth of coverage detected in whole genome (WG) data in non-repeated and coding regions across the whole genome assembly. The whole genome assembly was considered and not only the predicted ddRAD-seq regions.
