# Supplementary material for: Investigating unexplained genetic variation and its expression in the arbuscular mycorrhizal fungus Rhizophagus irregularis: A comparison of whole genome and RAD sequencing data
Source: PLoS One. 2019 Dec 27;14(12):e0226497. doi: 10.1371/journal.pone.0226497 (PMC6934306; doi:10.1371/journal.pone.0226497)
Supplement: S11 Fig — All positions in the genome, except the regions labelled as ‘repeats’, were considered for this analysis. Ran- domly chosen frequencies of one allele at di-allelic positions were considered in order to generate the distri- bution. Only positions with a total depth coverage higher or equal to the threshold were included. (PDF) [file pone.0226497.s012.pdf]

Figure S11: Allelic frequency graphs obtained with different depth coverage thresholds.
All positions in the genome, except the regions labelled as 'repeats', were considered for this analysis. Randomly chosen frequencies of one allele at di-allelic positions were considered in order to generate the distribution. Only positions with a total depth coverage higher or equal to the threshold were included.

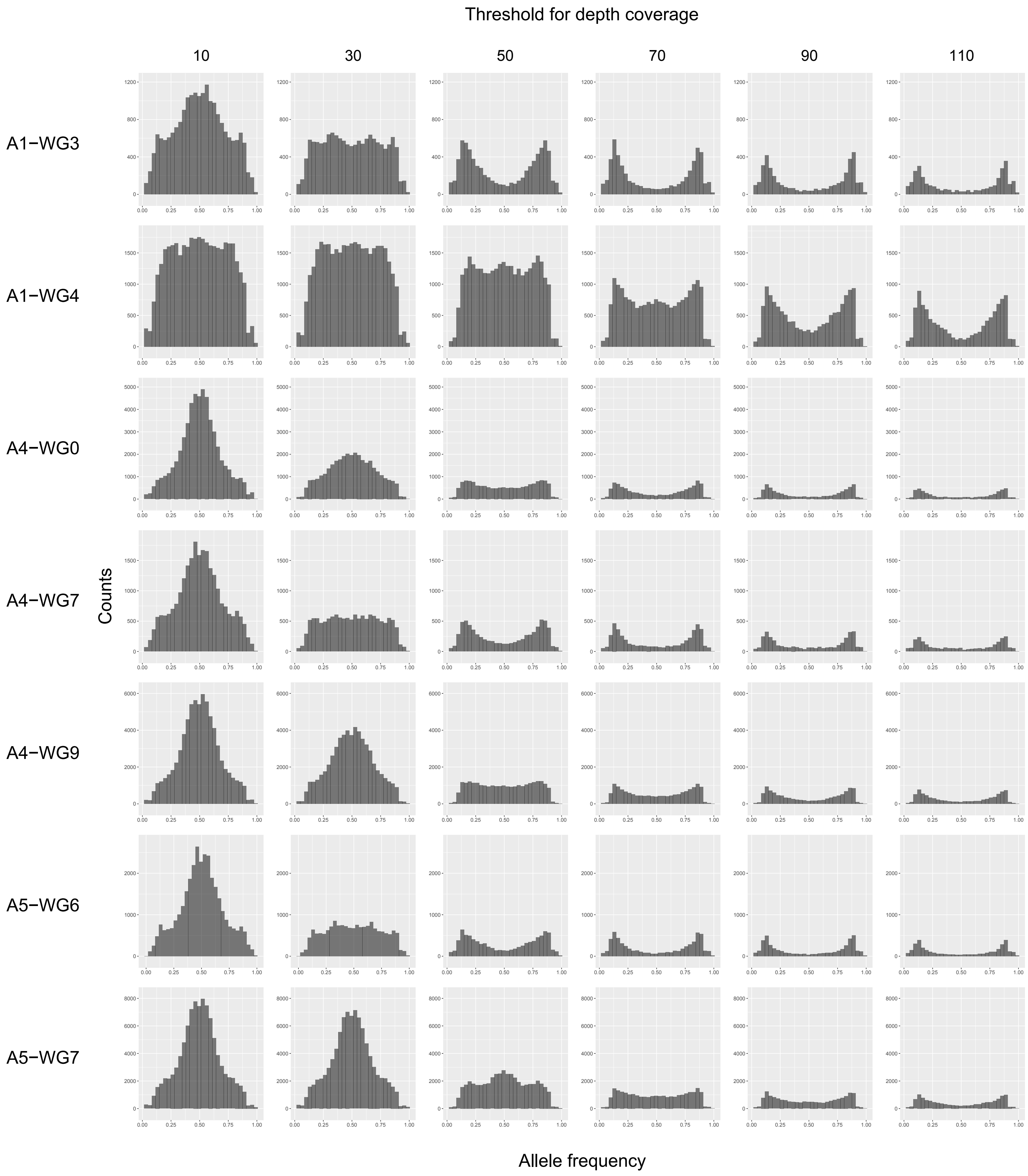

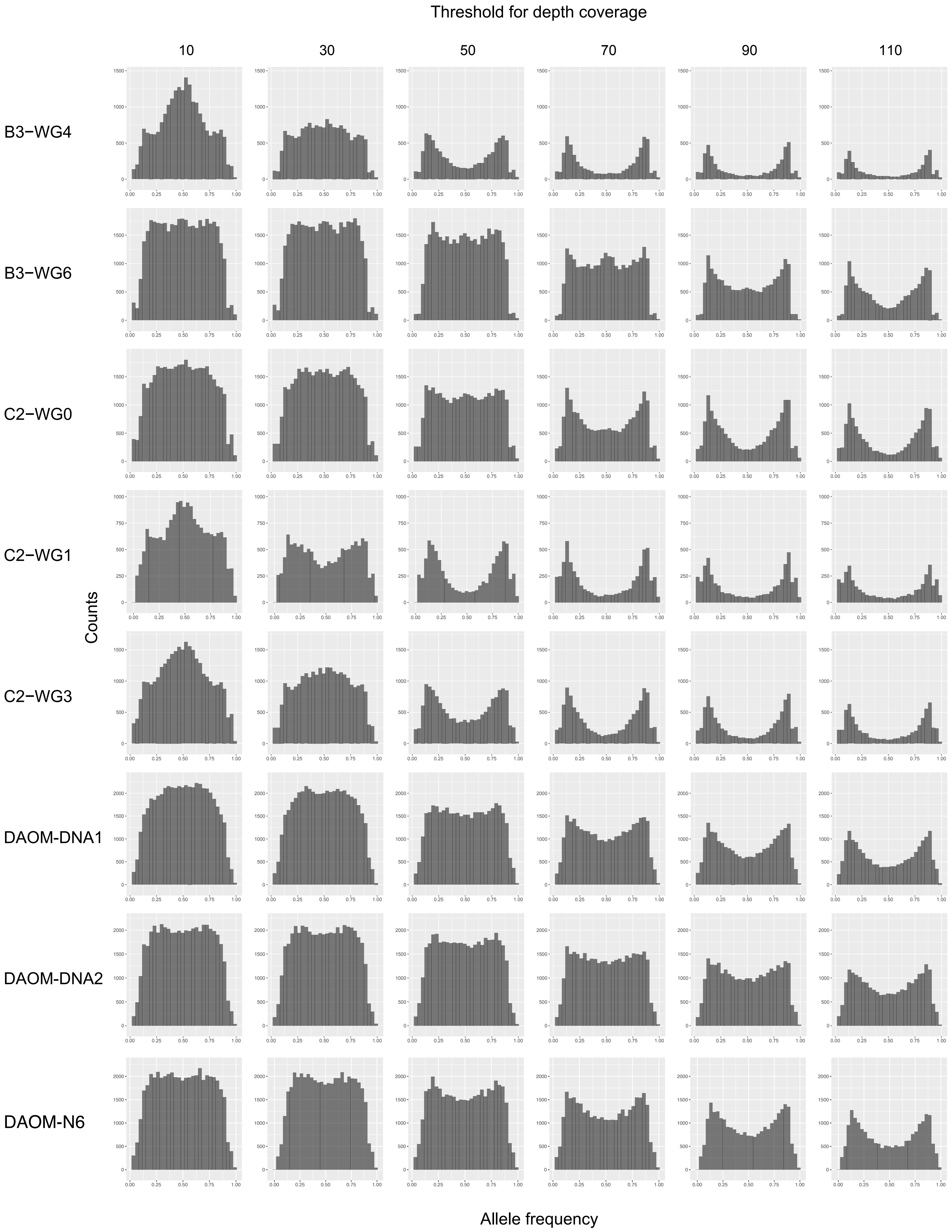

Threshold for depth coverage
10
30
50
70
90
110
B3−WG4
B3−WG6
C2−WG0
C2−WG1
C2−WG3
DAOM-DNA1
DAOM-DNA2
DAOM-N6
Counts
Allele frequency
