# Supplementary material for: Investigating unexplained genetic variation and its expression in the arbuscular mycorrhizal fungus Rhizophagus irregularis: A comparison of whole genome and RAD sequencing data
Source: PLoS One. 2019 Dec 27;14(12):e0226497. doi: 10.1371/journal.pone.0226497 (PMC6934306; doi:10.1371/journal.pone.0226497)
Supplement: S12 Fig — (PDF) [file pone.0226497.s013.pdf]

**Figure S12: Analysis of RNAseq data in other fungi and plants**

**a** **A. cinnamomea**

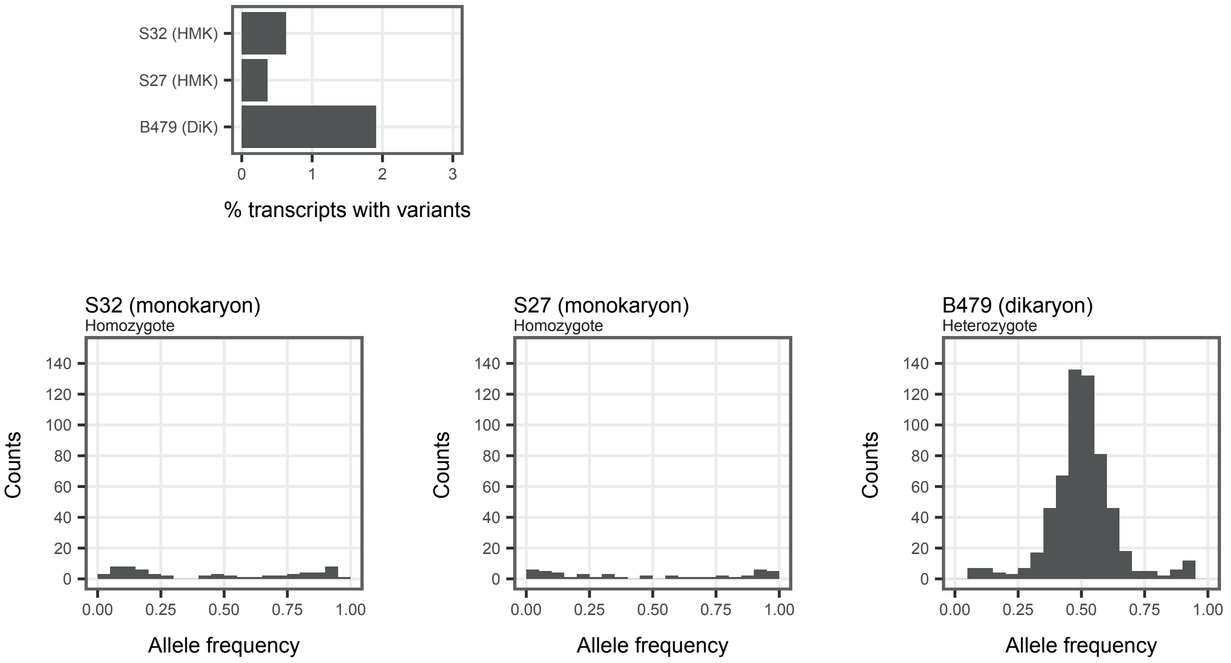


**b** **Arabidopsis**

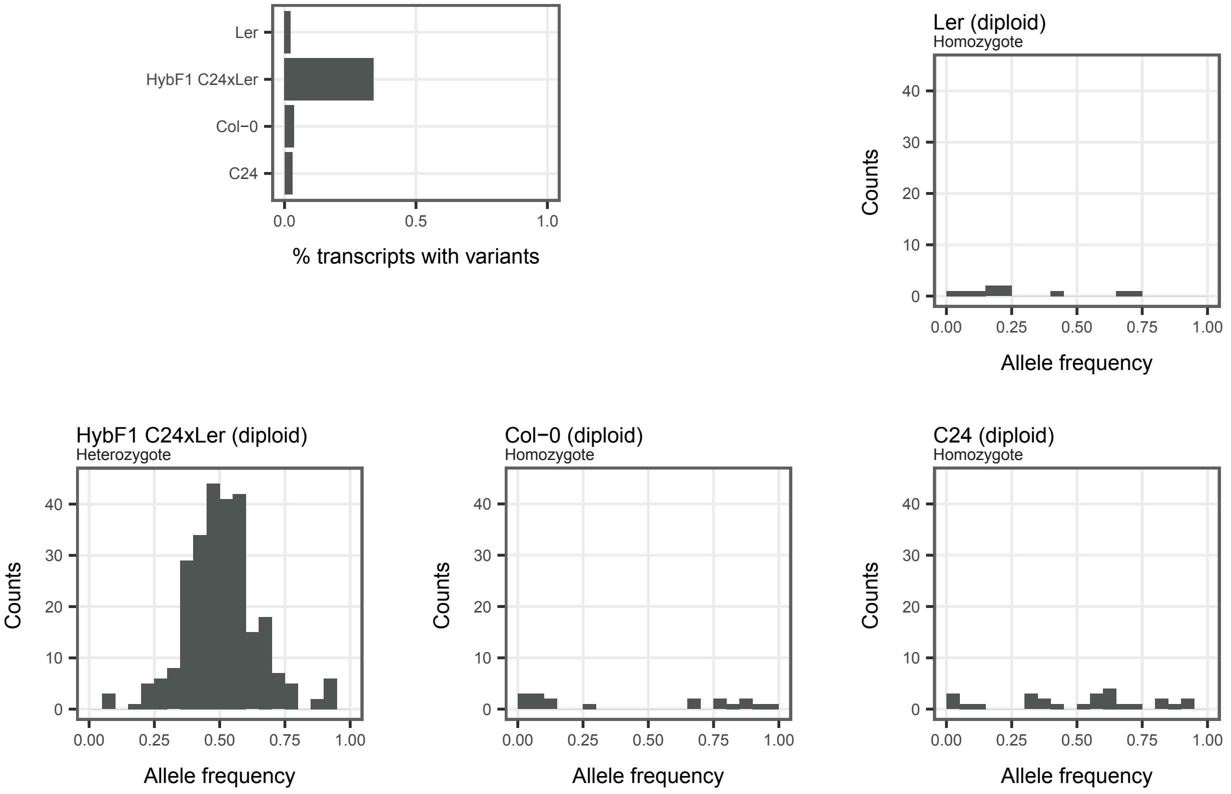

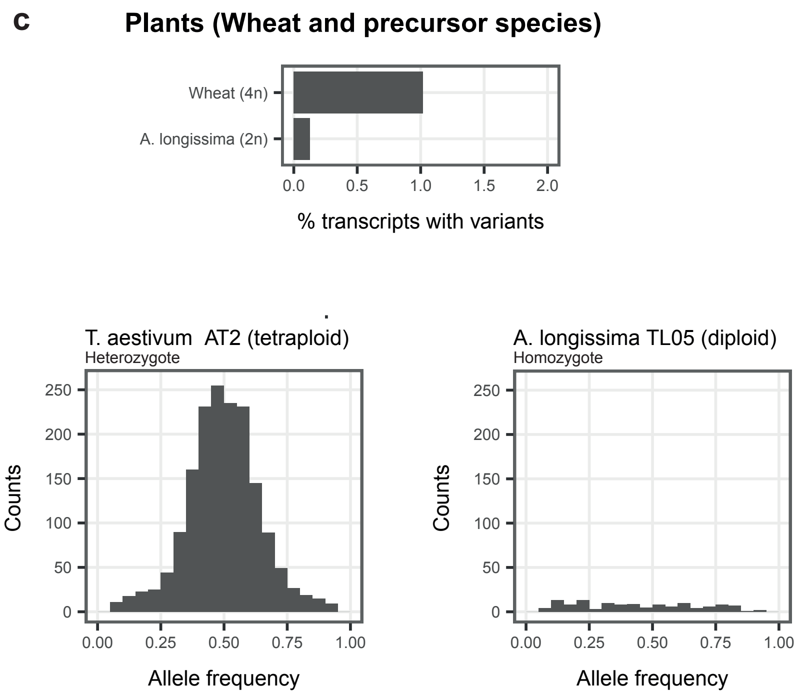
c
Plants (Wheat and precursor species)
Wheat (4n)
A. longissima (2n)
0.0
0.5
1.0
1.5
2.0
% transcripts with variants
T. aestivum AT2 (tetraploid)
Heterozygote
A. longissima TL05 (diploid)
Homozygote
Counts
0
50
100
150
200
250
0.00
0.25
0.50
0.75
1.00
Allele frequency

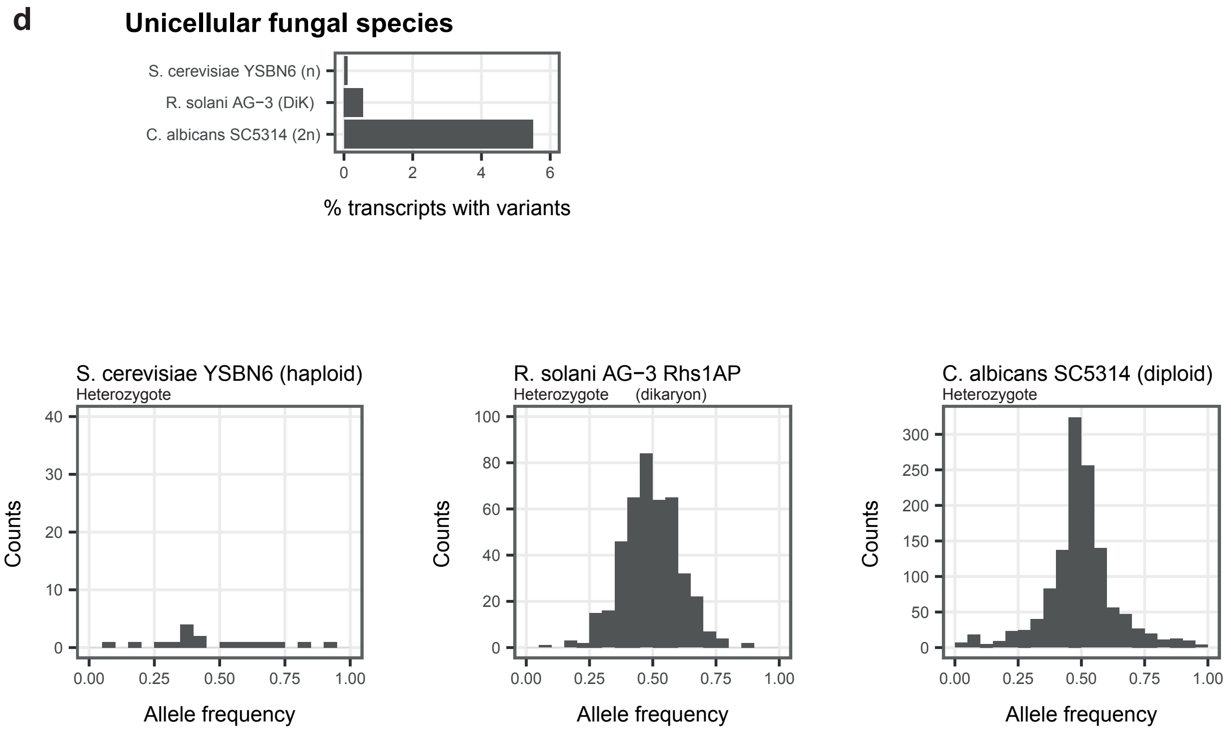
d
Unicellular fungal species
S. cerevisiae YSBN6 (n)
R. solani AG−3 (DiK)
C. albicans SC5314 (2n)
0
2
4
6
% transcripts with variants
S. cerevisiae YSBN6 (haploid)
Heterozygote
R. solani AG−3 Rhs1AP
Heterozygote (dikaryon)
C. albicans SC5314 (diploid)
Heterozygote
Counts
0
10
20
30
40
0
20
40
60
80
100
0
50
100
150
200
250
300
0.00
0.25
0.50
0.75
1.00
Allele frequency
