# Supplementary material for: Investigating unexplained genetic variation and its expression in the arbuscular mycorrhizal fungus Rhizophagus irregularis: A comparison of whole genome and RAD sequencing data
Source: PLoS One. 2019 Dec 27;14(12):e0226497. doi: 10.1371/journal.pone.0226497 (PMC6934306; doi:10.1371/journal.pone.0226497)
Supplement: S13 Fig — (a), (b) and (c) correspond to three examples in different regions of the genome displayed in IGV browser. Only nucleotides differing from the reference genome are shown with a colour different from light gray. Alleles contributing to bi-allelic positions in RNA-seq are surrounded with red circles or ellipses. (PDF) [file pone.0226497.s014.pdf]

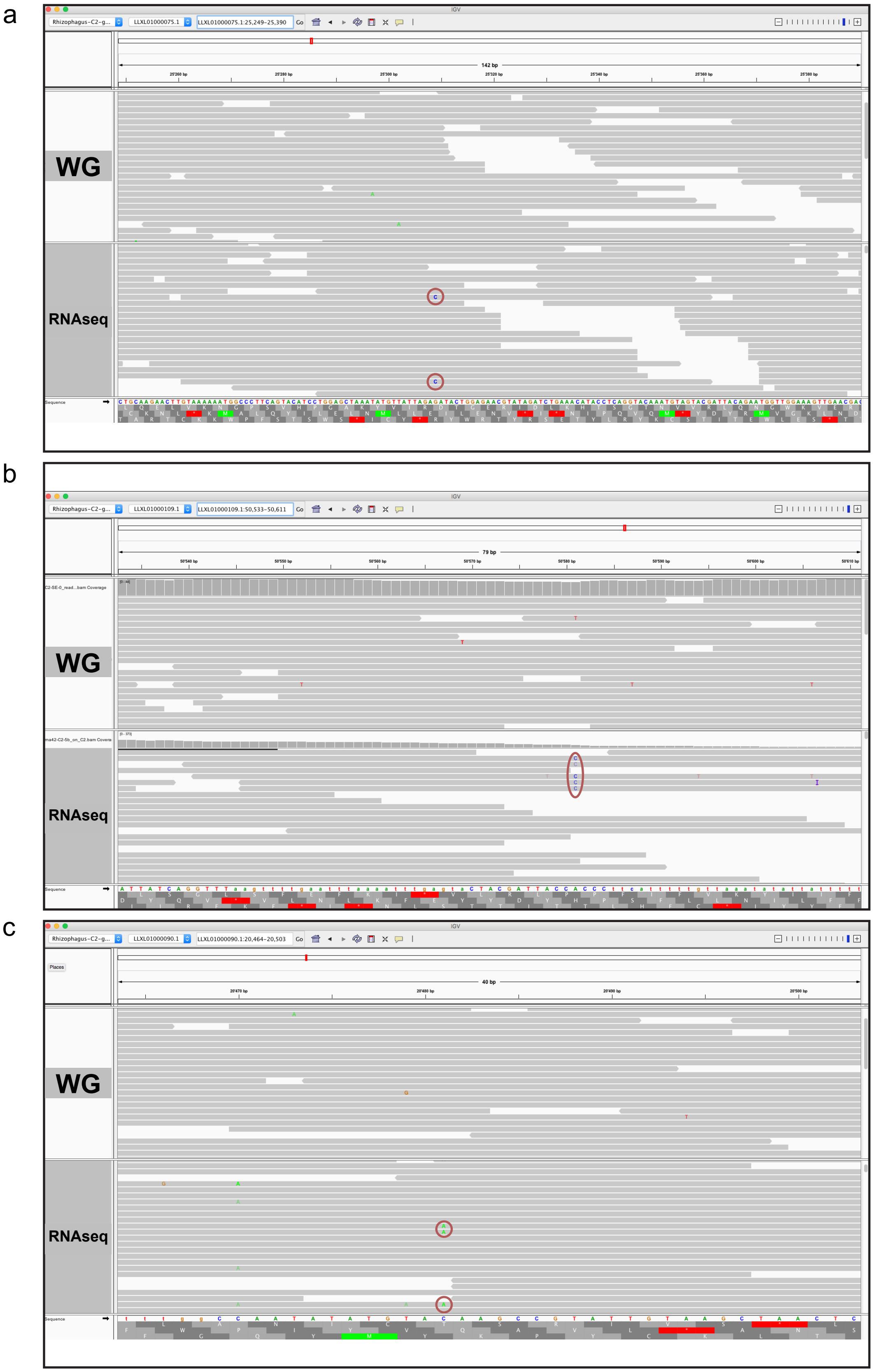

Figure S13: Examples of bi-allelic positions found in RNA-seq data and not in WG data in isolate C2. (a), (b) and (c) correspond to three examples in different regions of the genome displayed in IGV browser. Only nucleotides differing from the reference genome are shown with a colour different from light gray. Alleles contributing to bi-allelic positions in RNA-seq are surrounded with red circles or ellipses.
