# Supplementary material for: Investigating unexplained genetic variation and its expression in the arbuscular mycorrhizal fungus Rhizophagus irregularis: A comparison of whole genome and RAD sequencing data
Source: PLoS One. 2019 Dec 27;14(12):e0226497. doi: 10.1371/journal.pone.0226497 (PMC6934306; doi:10.1371/journal.pone.0226497)
Supplement: S15 Fig — (a), (b) and (c) correspond to three examples in different regions of the genome displayed in IGV browser. Colours other than light grey depict alleles different from the reference in the reads. ‘Depth’ shows a histogram of the depth of coverage for genomic reads. Interesting regions are labelled with red text and boxes. (PDF) [file pone.0226497.s016.pdf]

a
b
c

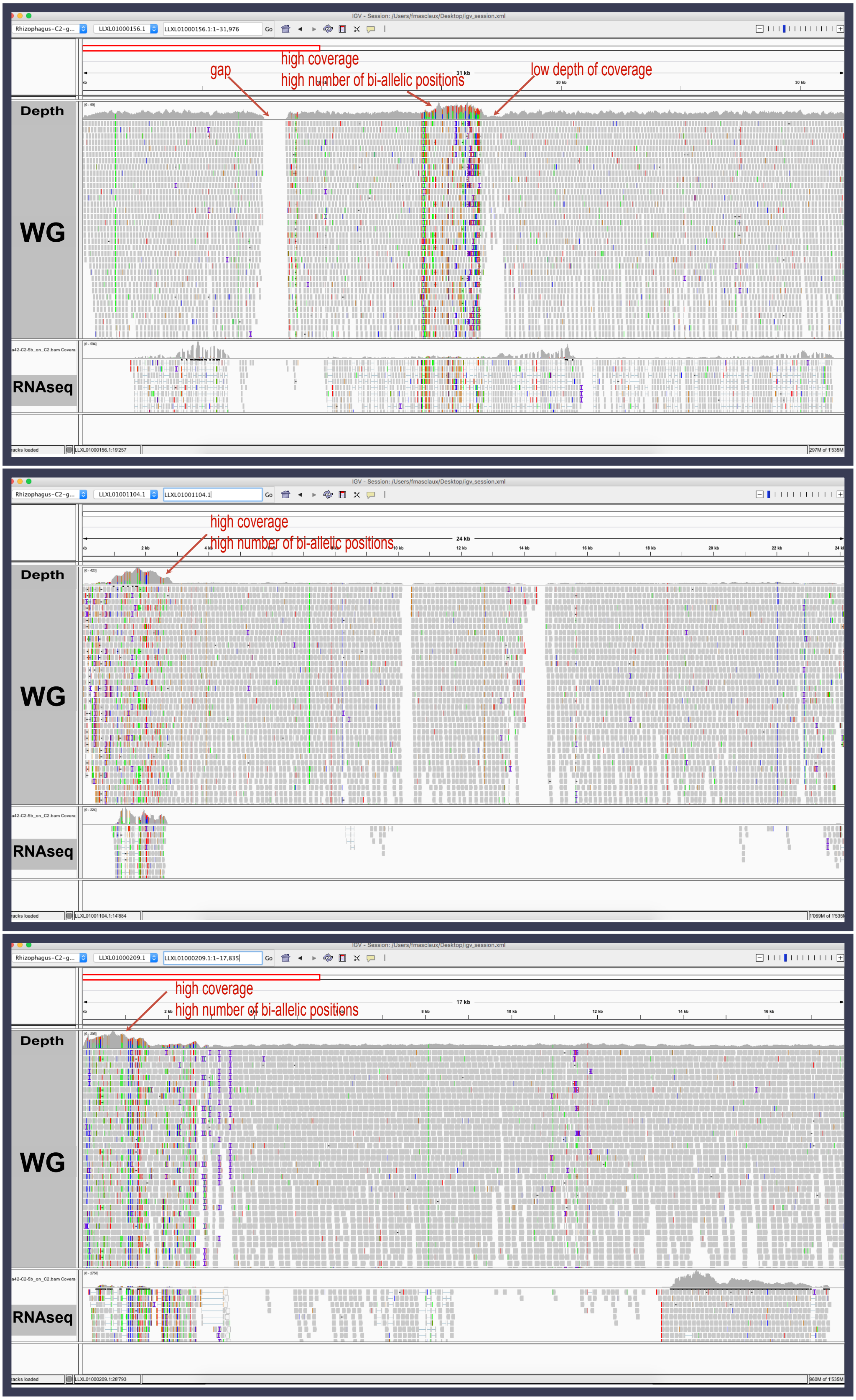

Figure S15: Examples of problematic regions in the genome assembly of the isolate C2. (a), (b) and (c) correspond to three examples in different regions of the genome displayed in IGV browser. Colours other than light grey depict alleles different from the reference in the reads. 'Depth' shows a histogram of the depth of coverage for genomic reads. Interesting regions are labelled with red text and boxes.
